# Identification and evaluation of reliable reference genes for quantitative real-time PCR analysis in tea plants under differential biotic stresses

**DOI:** 10.1038/s41598-020-59168-z

**Published:** 2020-02-12

**Authors:** Wei Xu, Yanan Dong, Yongchen Yu, Yuxian Xing, Xiwang Li, Xin Zhang, Xiangjie Hou, Xiaoling Sun

**Affiliations:** 1 College of Plant Protection, Jilin Agricultural University, Changchun, China; 2 Tea Research Institute, Chinese Academy of Agricultural Sciences, Hangzhou Zhejiang, China; 3 Key Laboratory of Tea Biology and Resources Utilization, Ministry of Agriculture, Hangzhou Zhejiang, China

**Keywords:** Plant stress responses, Biotic

## Abstract

The selection of reliable reference genes (RGs) for normalization under given experimental conditions is necessary to develop an accurate qRT-PCR assay. To the best of our knowledge, only a small number of RGs have been rigorously identified and used in tea plants (*Camellia sinensis* (L.) O. Kuntze) under abiotic stresses, but no critical RG identification has been performed for tea plants under any biotic stresses till now. In the present study, we measured the mRNA transcriptional levels of ten candidate RGs under five experimental conditions; these genes have been identified as stable RGs in tea plants. By using the ΔCt method, geNorm, NormFinder and BestKeeper, *CLATHRIN1* and *UBC1*, *TUA1* and *SAND1*, or *SAND1* and *UBC1* were identified as the best combination for normalizing diurnal gene expression in leaves, stems and roots individually; *CLATHRIN1* and *GAPDH1* were identified as the best combination for jasmonic acid treatment; *ACTIN1* and *UBC1* were identified as the best combination for *Toxoptera aurantii*-infested leaves; *UBC1* and *GAPDH1* were identified as the best combination for *Empoasca onukii*-infested leaves; and *SAND1* and *TBP1* were identified as the best combination for *Ectropis obliqua* regurgitant-treated leaves. Furthermore, our results suggest that if the processing time of the treatment was long, the best RGs for normalization should be recommended according to the stability of the proposed RGs in different time intervals when intragroup differences were compared, which would strongly increase the accuracy and sensitivity of target gene expression in tea plants under biotic stresses. However, when the differences of intergroup were compared, the RGs for normalization should keep consistent across different time points. The results of this study provide a technical guidance for further study of the molecular mechanisms of tea plants under different biotic stresses.

## Introduction

With the increasing popularity of gene expression analysis in biological research, quantitative real-time polymerase chain reaction (qRT-PCR) has become a critical and powerful tool for rapid and reliable quantification of mRNA transcriptional expression levels of target genes due to its high-throughput screening, sensitivity, simplicity, specificity and accuracy^1,2^. Relative quantification of target gene expression under certain stresses has been widely studied since the beginning of this century^3^. An accurate assay of gene expression through qRT-PCR relies on every step of sample preparation and processing, e.g., the integrity of purified RNA, the efficiency of reverse transcription, and the overall transcriptional activity of the tissues or cells analysed^4^; each step needs to be accurately normalized by stably expressed reference genes (RGs)^5,6^. Therefore, the selection of reliable RGs for normalization under given experimental conditions is a requirement for developing an accurate qPCR assay.

Housekeeping genes, such as the *glyceraldehyde 3-phosphate* (*GAPDH*), the *actin* gene (*ACTIN*), translation elongation factor *EF-1 alpha* (*EF-1α*), *18 s rRNA*, *25 S rRNA* and *poly-ubiquitin* (*UBQ*), have been commonly used as the normalization scalar in studies of relative quantification of plant target genes, some of which (*EF-1α*, *GAPDH*, *ACTIN*) have been identified as reliable RGs in certain plants under given experimental conditions^7–10^. However, to date, no RG has been found to exhibit perfectly stable expression in all plant species, even in the same tissue from the same plant species, but under different experimental conditions^11–13^. For instance, *DcACTIN* and *DcUBQ* have been identified as the top two stable RGs in carrot (*Daucus carota* L.) under abiotic stresses, but *eIF-4α* and *GAPDH* have been ranked in the top two RGs in carrots under hormone stimuli^7^; in tea plants (*Camellia sinensis* (L.) O. Kuntze), *CsTIP41* was identified as the most stable RG for leaf development, but *CsTBP* was identified as the most stable RG for tea leaves under hormone stimuli^14^. Therefore, to avoid missing or overemphasizing potential biological changes of target gene expression, it is essential to identify optimum stable RGs for the proposed research object, for different tissues of the same species, for the same tissue of the same species under different biotic or abiotic stresses and their processing time.

Tea is one of the most important leaf-type woody cash crops in China, and the tender buds and leaves of this plant are the raw material for commercial tea. Since the publication of the draft genome sequence of *C. sinensis* var. *sinensis*^15^, the molecular mechanisms of aroma components biosynthesis, cold spells or resistance, drought resistance, barren tolerance, and other interactions of tea plants with environmental factors or with other organisms around them have been elucidated^16–20^. During the development of tea plant, it usually suffers serious damage from the infestation of insect herbivores all year round. Therefore, the chemical and molecular mechanisms under interactions between tea plants and their herbivorous pests need to be widely excavated to offer theoretical foundations for utilizing chemical signals between them to control tea pests or breeding new insect-resistant tea varieties. The RGs used previously in the studies of herbivores (*Ectropis obliqua*, *Empoasca onukii*) induced tea plant defensive responses at the gene transcriptional level, such as *CsGAPDH* and *18SrRNA*^21–23^, were roughly selected from previously reported RGs without critical identification under given experimental conditions, which may lead to the deviation of the results to some extent and may also lead to the neglect of some important experimental phenomena. Therefore, it is important to define the RG for qRT-PCR analysis in tea plants under infestations of different pests and their related biotic stresses.

According to previous reports, *CsACTIN1*, Clathrin adaptor complex subunit (*CsCLATHRIN1*), *CsEF1*, *CsGAPDH1*, SAND family protein gene (*CsSAND1*), Tap42-interacting protein of 41 kDa (*CsTIP41*), Ubiquitin-conjugating enzyme (*CsUBC1*), Polypyrimidine tract-binding protein (*CsPTB1*), alpha-1 tubulin (*CsTUA1*) and TATA-box binding protein gene (*CsTBP1*) are frequently used as stable RGs in the process of mRNA expression analysis (Tables 1 and 2)^20,24–29^. In the present study, we measured mRNA transcriptional levels of the above mentioned ten RGs in different tissues of tea plants in circadian rhythms, jasmonic acid-treated tea leaves, *T. aurantii* infested tea leaves, *E. onukii* infested tea leaves, and tea leaves treated with mechanical damage plus *E. obliqua* regurgitant. The results were evaluated by BestKeeper, geNorm, NormFinder and the ΔCt method to identify the most stably expressed RGs firstly; secondly, RefFinder was used to integrate the results to determine the most stable RG for each treatment. Finally, to demonstrate the importance of stable RGs in the normalization process of tea plants under infestations of different pests or their related biotic stresses, *CsMYC2*, *CsOPR3*, *CsPAL* and *CsPALc* were chosen as the target genes for validation. As we all know, *MYC2* was a key transcription factor of JA signaling pathway^30^; OPR3 is the isoenzyme relevant for JA biosynthesis^22^ and *PAL* were closely associated with the accumulation of endogenous SA^31^. The aim of this study was to select the most appropriate RGs for the gene expression analysis of tea plants under different biotic stresses.

**Table 1:** Ten housekeeping genes frequently used for qRT-PCR of tea plant.

NO.	Abbreviation	Given conditions	Ref.
1	*CsACTIN1*	Different organs Nitrogen stress Fe stress	Sun *et al*.^29^; Liu *et al*.^20^; Wang *et al*.^24^
2	*CsCLATHRIN1*	Different organs Leaves with Cold and short photoperiod treatments Shoots after auxin antagonist auxinole treatments	Hao *et al*.^28^
3	*CsEF1*	Diurnal expression in leaves	Hao *et al*.^28^
4	*CsGAPDH1*	Different maturity of leaves Leaves with Cold and drought treatments Nitrogen stress Drought, cold, Al, and NaCl stresses	Sun *et al*.^29^; Ma *et al*.^25^; Liu *et al*.^20^
5	*CsSAND1*	Different organs	Hao *et al*.^28^
6	*CsTIP41*	In various tea leaf developmental stages	Wu *et al*.^26^
7	*CsUBC1*	Shoots with cold and short photoperiod treatments Mn stress	Hao *et al*.^28^; Wang *et al*.^24^
8	*CsPTB1*	Shoots after auxin antagonist auxinole treatment	Hao *et al*.^28^
9	*CsTUA1*	Physical damages	Ma *et al*.^25^
10	*CsTBP*	In various tea leaf developmental stages Leaves with hormone treatments Mn stress Post-harvest leaves Posharvest	Wu *et al*.^26^; Wang *et al*.^24^; Zhou *et al*.^27^

**Table 2:** Sequence Information of the Candidate Reference Genes and Target Genes.

Name	GeneBank Accession Number	Primer sequence (5′–3′) forward/reverse	Amplicon Length (bp)	qRT-PCR Efficiency (%)
*CsEF1*	KA280301.1	TTGGACAAGCTCAAGGCTGAACG	110	98
		ATGGCCAGGAGCATCAATGACAGT		
*CsCLATHRIN1*	KA291473.1	TAGAGCGGGTAGTGGAGACCTCGTT	129	102
		TACCAAAGCCGGCTCGTATGAGATT		
*CsACTIN1*	KA280216.1	TGGGCCAGAAAGATGCTTATGTAGG	118	103
		ATGCCAGATCTTTTCCATGTCATCC		
*CsGAPDH1*	KA295375.1	TTTTTGGCCTTAGGAACCCAGAGG	107	93
		GGGCAGCAGCCTTATCCTTATCAGT		
*CsSAND1*	KM057790	TCCAATTGCCCCCTTAATGACTCA	109	106
		GTAAGGGCAGGCAAACACCAGGTA		
*CsTIP41*	AT4G34270	TGGAGTTGGAAGTGGACGAGACCGA	176	103.6
		CTCTGGAAAGTGGGATGTTTGAAGC		
*CsUBC1*	KA281185.1	TGCTGGTGGGGTTTTTCTTGTTACC	124	92
		AAGGCATATGCTCCCATTGCTGTTT		
*CsPTB1*	GAAC01052498.1	TGACCAAGCACACTCCACACTATCG	107	95
		TGCCCCCTTATCATCATCCACAA		
*CsTUA1*	JN399223.1	TCACTGTTTACCCATCTCCC	167	106.1
		GTAGGTGGGTCGCTCAATAT		
*CsTBP*	AT1G55520	GGCGGATCAAGTGTTGGAAGGGAG	166	107.0
		ACGCTTGGGATTGTATTCGGCATTA		
*CsMYC2*	EF645810	TAGCGGTTGTGGCGGAGATT		
		TGAGCTTCTCTCGCCTCTGC		
*CsOPR3*	XM_028243785.1	CGATCAACAGCCGGTGGATTT		
		GCGTGGACAGCATCAACCAC		
*CsPAL*	D26596.1	CCAATTCCTTGCCAATCCTGTAAC		
		CAACTGCCTCGGCTGTCTTTCT		
*CsPALc*	KY615671	CGGAACAAGGCCTTACATGG		
		TGGGCAAACATGAGCTTTCC		

## Results

### Expression profiles of candidate reference genes

**Figure 1:**
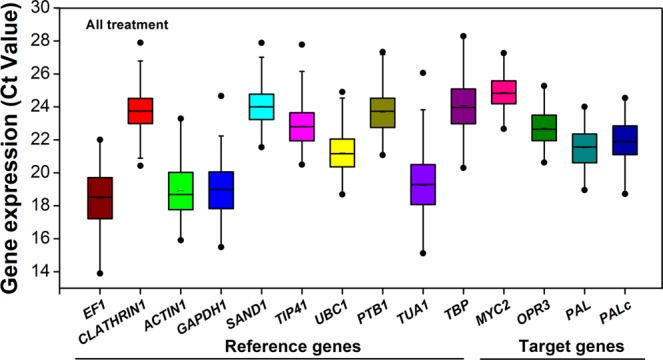
Expression Profiles of Ten Candidate Reference Genes and Four Target Genes in *C. sinensis*. The expression level of RGs in all samples is performed in terms of the cycle threshold number (Ct value). The data are expressed as box-whisker plots; the short bar in the box refers to the Ct mean value; the box represents the 25th–75th percentiles; the median is indicated by a bar across the box; the whiskers on each box represent the distribution of the Ct values; and the dark spots refer to extreme outliers.

The expression level of RGs in all treatments is performed in terms of the cycle threshold number (Ct value). As shown in Fig. 1, the raw Ct values of all candidate RGs ranged from 13.90 (*EF1*) to 28.29 (*TBP*). *EF1* (18.44), *ACTIN1* (18.91), *GAPDH1* (18.97) and *TUA1* (19.23) were the most abundant transcripts, reaching the threshold fluorescence peak after 18 cycles. *PTB1* (23.65), *CLATHRIN1* (23.71), *SAND1* (24.04) and *TBP* (24.08) were expressed at the lowest levels. The raw Ct values of the four target genes ranged from 18.72 (*PALc*) to 27.26 (*MYC2*). More details were shown in Fig. S8.

### Diurnal expression in different tissues

#### Leaf

**Figure 2:**
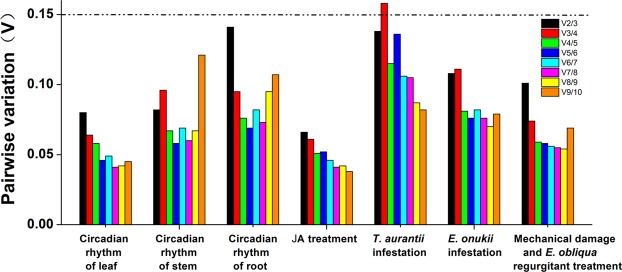
Optimal Number of Reference Genes for the Normalization of *C. sinensis* under Different Experimental Manipulations. The pairwise variation (Vn/n + 1) was analysed by geNorm software to determine the optimal number of RGs included in the qPCR analysis. Values less than 0.15 indicate that another RG will not significantly improve normalization.

The gene expression stability of ten candidate RGs for leaves with circadian rhythm was analyzed by using geNorm, NormFinder, BestKeeper and the ΔCt method. The results showed that the gene stability ranking as analyzed by BestKeeper differed from the ranking as analyzed by the other three methods. For example, geNorm, NormFinder and the ΔCt method identified *UBC1* and *CLATHRIN1* as the most stable 2 of the 10 RGs in all test periods (from 0:00 am to 22:00 pm), whereas BestKeeper identified *GAPDH1* and *CLATHRIN1* as the most stable 2 of the 10 RGs for diurnal expression in leaves. However, all four methods identified *PTB1* as the most variable RG. According to the results from RefFinder, the stability ranking of RGs from the most to the least was as follows: *UBC1* > *CLATHRIN1* > *GAPDH1* > *TBP* > *EF1* > *SAND1* > *TUA1* > *ACTIN1* > *TIP41* > *PTB1* (Table 3). With GeNorm (Fig. 2), all pairwise variation (Vn/n + 1) was below 0.15 (the recommended cut-off), indicating that the inclusion of an additional RG was unnecessary. Based on the ranking of the RGs by RefFinder, *CLATHRIN1* and *UBC1* were identified as the best combination for normalizing the diurnal expression in leaves (Tables 4 and 5).

**Table 3:** Ranking of 10 Reference Genes Expression under Different Experimental Manipulations.

Group	Rank	geNorm		NormFinder		BestKeeper			ΔCt		RefFinder
		Reference Gene	Stability	Reference Gene	Stability	Reference Gene	Standard Deviation	*r*	Reference Gene	Standard Deviation	
Circadian rhythm of leaf	1	*UBC*	0.243	*UBC1*	0.160	*GAPDH1*	0.366	0.885	*UBC1*	0.333	*UBC1*
	2	*CLATHRIN1*	0.243	*CLATHRIN1*	0.201	*CLATHRIN1*	0.367	0.894	*CLATHRIN1*	0.353	*CLATHRIN1*
	3	*TBP*	0.267	*GAPDH1*	0.225	*ACTIN1*	0.383	0.726	*GAPDH1*	0.362	*GAPDH1*
	4	*GAPDH1*	0.284	*TBP*	0.256	*UBC1*	0.391	0.933	*TBP*	0.379	*TBP*
	5	*EF1*	0.308	*SAND1*	0.274	*TBP*	0.396	0.858	*SAND1*	0.395	*EF1*
	6	*TUA1*	0.320	*TUA1*	0.288	*EF1*	0.417	0.863	*EF1*	0.401	*SAND1*
	7	*SAND1*	0.343	*EF1*	0.289	*TUA1*	0.442	0.868	*TUA1*	0.402	*TUA*
	8	*TIP41*	0.357	*TIP41*	0.296	*SAND1*	0.469	0.891	*TIP41*	0.408	*ACTIN1*
	9	*ACTIN1*	0.373	*ACTIN1*	0.373	*TIP41*	0.486	0.871	*ACTIN1*	0.455	*TIP41*
	10	*PTB1*	0.399	*PTB1*	0.434	*PTB1*	0.583	0.858	*PTB1*	0.503	*PTB1*
Circadian rhythm of stem	1	*SAND1*	0.208	*TUA1*	0.184	*UBC1*	0.241	0.559	*TUA1*	0.492	*TUA1*
	2	*TIP41*	0.208	*CLATHRIN1*	0.253	*TUA1*	0.264	0.819	*CLATHRIN1*	0.525	*SAND1*
	3	*PTB1*	0.246	*SAND1*	0.315	*SAND1*	0.270	0.547	*SAND1*	0.532	*CLATHRIN1*
	4	*UBC1*	0.323	*ACTIN1*	0.33	*CLATHRIN1*	0.328	0.792	*TIP41*	0.548	*UBC1*
	5	*TUA1*	0.347	*UBC1*	0.334	*TIP41*	0.331	0.577	*UBC1*	0.552	*TIP41*
	6	*CLATHRIN1*	0.368	*TIP41*	0.342	*PTB1*	0.342	0.530	*PTB1*	0.574	*PTB1*
	7	*ACTIN1*	0.410	*PTB1*	0.375	*TBP*	0.377	0.786	*ACTIN1*	0.591	*ACTIN1*
	8	*TBP*	0.443	*TBP*	0.376	*ACTIN1*	0.467	0.869	*TBP*	0.604	*TBP*
	9	*EF1*	0.490	*EF1*	0.599	*EF1*	0.520	0.615	*EF1*	0.733	*EF1*
	10	*GAPDH1*	0.639	*GAPDH1*	1.182	*GAPDH1*	0.768	0.719	*GAPDH1*	1.234	*GAPDH1*
Circadian rhythm of root	1	*SAND1*	0.308	*UBC1*	0.211	*TIP41*	0.431	0.833	*UBC1*	0.581	*SAND1*
	2	*TBP*	0.308	*SAND1*	0.287	*CLATHRIN1*	0.433	0.851	*SAND1*	0.594	*UBC1*
	3	*TIP41*	0.367	*CLATHRIN1*	0.323	*SAND1*	0.454	0.878	*TBP*	0.609	*TBP*
	4	*CLATHRIN1*	0.421	*TBP*	0.327	*PTB1*	0.471	0.738	*CLATHRIN1*	0.617	*TIP41*
	5	*UBC1*	0.429	*TIP41*	0.349	*UBC1*	0.492	0.931	*TIP41*	0.618	*CLATHRIN1*
	6	*PTB1*	0.451	*PTB1*	0.459	*TBP*	0.520	0.909	*PTB1*	0.680	*PTB1*
	7	*GAPDH1*	0.502	*GAPDH1*	0.496	*EF1*	0.616	0.800	*GAPDH1*	0.710	*GAPDH1*
	8	*EF1*	0.549	*EF1*	0.584	*GAPDH1*	0.660	0.939	*EF1*	0.780	*EF1*
	9	*TUA1*	0.638	*TUA1*	0.885	*ACTIN1*	0.814	0.387	*TUA1*	0.995	*TUA1*
	10	*ACTIN1*	0.727	*ACTIN1*	0.987	*TUA1*	0.992	0.857	*ACTIN1*	1.085	*ACTIN1*
JA treatment	1	*CLATHRIN1*	0.209	*CLATHRIN1*	0.132	*SAND1*	0.194	0.604	*CLATHRIN1*	0.290	*CLATHRIN1*
	2	*GAPDH1*	0.209	*GAPDH1*	0.166	*PTB1*	0.194	0.42	*GAPDH1*	0.303	*GAPDH1*
	3	*UBC1*	0.221	*UBC1*	0.213	*TIP41*	0.196	0.625	*UBC1*	0.325	*UBC1*
	4	*SAND1*	0.250	*TIP41*	0.228	*GAPDH1*	0.223	0.815	*TIP41*	0.333	*TIP41*
	5	*TIP41*	0.269	*TBP*	0.231	*UBC1*	0.227	0.716	*TBP*	0.340	*PTB1*
	6	*PTB1*	0.281	*ACTIN1*	0.234	*CLATHRIN1*	0.269	0.893	*ACTIN1*	0.342	*SAND1*
	7	*ACTIN1*	0.297	*SAND1*	0.243	*ACTIN1*	0.322	0.876	*SAND1*	0.346	*TBP*
	8	*TBP*	0.309	*PTB1*	0.313	*TBP*	0.332	0.864	*PTB1*	0.384	*ACTIN1*
	9	*EF1*	0.329	*EF1*	0.325	*EF1*	0.379	0.868	*EF1*	0.400	*EF1*
	10	*TUA1*	0.349	*TUA1*	0.363	*TUA1*	0.421	0.796	*TUA1*	0.432	*TUA1*
*T. aurantii* infestation	1	*ACTIN1*	0.490	*ACTIN1*	0.336	*ACTIN1*	0.32	0.501	*ACTIN1*	0.709	*ACTIN1*
	2	*TBP*	0.490	*UBC1*	0.515	*EF1*	0.412	0.184	*UBC1*	0.777	*UBC1*
	3	*CLATHRIN1*	0.507	*GAPDH1*	0.563	*GAPDH1*	0.458	0.553	*GAPDH1*	0.812	*GAPDH1*
	4	*GAPDH1*	0.531	*CLATHRIN1*	0.592	*UBC1*	0.464	0.510	*CLATHRIN1*	0.820	*CLATHRIN1*
	5	*TIP41*	0.541	*EF1*	0.617	*CLATHRIN1*	0.465	0.453	*PTB1*	0.848	*TBP*
	6	*UBC1*	0.688	*PTB1*	0.639	*TBP*	0.533	0.456	*EF1*	0.855	*EF1*
	7	*SAND1*	0.758	*SAND1*	0.643	*PTB*	0.560	0.617	*SAND1*	0.869	*PTB1*
	8	*PTB1*	0.792	*TBP*	0.682	*SAND1*	0.571	0.558	*TBP*	0.872	*SAND1*
	9	*EF1*	0.815	*TIP41*	0.756	*TIP41*	0.638	0.508	*TIP41*	0.914	*TIP41*
	10	*TUA1*	0.843	*TUA1*	0.792	*TUA1*	0.65	0.441	*TUA*	0.954	*TUA1*
*E. onukii* infestation	1	*GAPDH1*	0.275	*UBC1*	0.201	*EF1*	0.560	0.892	*UBC1*	0.574	*UBC1*
	2	*UBC1*	0.275	*GAPDH1*	0.230	*GAPDH1*	0.590	0.941	*GAPDH1*	0.585	*GAPDH1*
	3	*EF1*	0.334	*TIP41*	0.338	*CLATHRIN1*	0.620	0.761	*EF1*	0.628	*EF1*
	4	*TIP41*	0.420	*EF1*	0.347	*TIP41*	0.630	0.891	*TIP41*	0.643	*TIP41*
	5	*SAND1*	0.461	*SAND1*	0.439	*SAND1*	0.660	0.868	*SAND1*	0.688	*SAND1*
	6	*TBP*	0.491	*TBP*	0.466	*UBC1*	0.660	0.957	*TBP*	0.701	*CLATHRIN1*
	7	*TUA1*	0.542	*TUA1*	0.566	*PTB1*	0.700	0.494	*TUA1*	0.773	*TBP*
	8	*CLATHRIN1*	0.583	*CLATHRIN1*	0.589	*ACTIN1*	0.730	0.715	*CLATHRIN1*	0.784	*TUA1*
	9	*ACTIN1*	0.664	*ACTIN1*	0.868	*TBP*	0.800	0.924	*ACTIN1*	0.995	*ACTIN1*
	10	*PTB1*	0.743	*PTB1*	0.947	*TUA1*	0.860	0.894	*PTB1*	1.058	*PTB1*
Mechanical damage and *E.obliqua* regurgitant treatment	1	*SAND1*	0.261	*SAND1*	0.194	*ACTIN1*	0.344	0.806	*SAND1*	0.422	*SAND1*
	2	*TBP*	0.322	*TBP*	0.216	*CLATHRIN1*	0.372	0.799	*TBP*	0.435	*TBP*
	3	*CLATHRIN1*	0.337	*PTB1*	0.240	*TBP*	0.381	0.897	*PTB1*	0.451	*CLATHRIN1*
	4	*TIP41*	0.343	*CLATHRIN1*	0.279	*PTB1*	0.382	0.862	*CLATHRIN1*	0.460	*PTB1*
	5	*PTB1*	0.363	*ACTIN1*	0.292	*SAND1*	0.429	0.915	*TIP41*	0.477	*ACTIN1*
	6	*UBC1*	0.388	*TIP41*	0.328	*TIP41*	0.436	0.810	*ACTIN1*	0.482	*TIP41*
	7	*ACTIN1*	0.420	*UBC1*	0.374	*UBC1*	0.447	0.801	*UBC1*	0.513	*UBC1*
	8	*EF1*	0.453	*EF1*	0.451	*EF1*	0.494	0.698	*EF1*	0.576	*EF1*
	9	*GAPDH1*	0.518	*GAPDH1*	0.460	*GAPDH1*	0.520	0.779	*GAPDH1*	0.583	*GAPDH1*
	10	*TUA1*	0.261	*TUA1*	0.709	*TUA1*	0.616	0.537	*TUA1*	0.775	*TUA1*

**Table 4:** Ranking of 10 Reference Genes Expression in Different Processing Time under Different Experimental Manipulations.

Analysis Tool	Ranking Order (from the most stable to the least stable)									
	1	2	3	4	5	6	7	8	9	10
**JA treatment in the time interval from 0.5 h to 1.5 h**										
ΔCT	*CLATHRIN1*	*UBC1*	*ACTIN1*	*TIP41*	*TBP*	*GAPDH1*	*PTB1*	*EF1*	*SAND1*	*TUA1*
BestKeeper	*TIP41*	*PTB1*	*CLATHRIN1*	*UBC1*	*SAND1*	*GAPDH1*	*TBP*	*ACTIN1*	*EF1*	*TUA1*
Normfinder	*CLATHRIN1*	*UBC1*	*ACTIN1*	*TIP41*	*TBP*	*SAND1*	*GAPDH1*	*PTB1*	*EF1*	*TUA1*
Genorm	*CLATHRIN1 | UBC1*	*ACTIN1*	*GAPDH1*	*EF1*	*TIP41*	*TBP*	*PTB1*	*SAND1*	*TUA1*	
Recommended comprehensive ranking	*CLATHRIN1*	*UBC1*	*TIP41*	*ACTIN1*	*PTB1*	*GAPDH1*	*TBP*	*SAND1*	*EF1*	*TUA1*
**JA treatment in the time interval from 3 h to 6 h**										
ΔCT	*GAPDH1*	*UBC1*	*TIP41*	*CLATHRIN1*	*TBP*	*PTB1*	*SAND1*	*EF1*	*TUA1*	*ACTIN1*
BestKeeper	*TBP*	*SAND1*	*GAPDH1*	*PTB1*	*UBC1*	*TIP41*	*CLATHRIN1*	*EF1*	*TUA1*	*ACTIN1*
Normfinder	*GAPDH1*	*UBC1*	*TIP41*	*CLATHRIN1*	*TBP*	*PTB1*	*SAND1*	*EF1*	*TUA1*	*ACTIN1*
Genorm	*TIP41 | PTB1*	*CLATHRIN1*	*UBC1*	*GAPDH1*	*TBP*	*SAND1*	*EF1*	*TUA1*	*ACTIN1*	
Recommended comprehensive ranking	*GAPDH1*	*TIP41*	*UBC1*	*PTB1*	*TBP*	*CLATHRIN1*	*SAND1*	*EF1*	*TUA1*	*ACTIN1*
**JA treatment in the time interval from 12 h to 48 h**										
ΔCT	*CLATHRIN1*	*TBP*	*GAPDH1*	*ACTIN1*	*SAND1*	*TIP41*	*EF1*	*UBC1*	*TUA1*	*PTB1*
BestKeeper	*CLATHRIN1*	*SAND1*	*GAPDH1*	*UBC1*	*TIP41*	*PTB1*	*TBP*	*ACTIN1*	*TUA1*	*EF1*
Normfinder	*CLATHRIN1*	*TBP*	*GAPDH1*	*ACTIN1*	*TIP41*	*SAND1*	*EF1*	*UBC1*	*TUA1*	*PTB1*
Genorm	*CLATHRIN1 | GAPDH1*	*TBP*	*ACTIN1*	*SAND1*	*EF1*	*UBC1*	*TIP41*	*TUA1*	*PTB1*	
Recommended comprehensive ranking	*CLATHRIN1*	*GAPDH1*	*TBP*	*SAND1*	*ACTIN1*	*TIP41*	*UBC1*	*EF1*	*PTB1*	*TUA1*
***T. aurantii*** **infestation in the time interval from 6 h to 24 h**										
ΔCT	*ACTIN1*	*UBC1*	*GAPDH1*	*CLATHRIN1*	*TBP*	*SAND1*	*PTB1*	*EF1*	*TIP41*	*TUA1*
BestKeeper	*ACTIN1*	*CLATHRIN1*	*UBC1*	*GAPDH1*	*EF1*	*TBP*	*SAND1*	*PTB1*	*TIP41*	*TUA1*
Normfinder	*ACTIN1*	*UBC1*	*GAPDH1*	*CLATHRIN1*	*SAND1*	*EF1*	*TBP*	*PTB1*	*TIP41*	*TUA1*
Genorm	*ACTIN1 | TBP*	*CLATHRIN1*	*TIP41*	*GAPDH1*	*UBC1*	*SAND1*	*PTB1*	*EF1*	*TUA1*	
Recommended comprehensive ranking	*ACTIN1*	*UBC1*	*CLATHRIN1*	*GAPDH1*	*TBP*	*SAND1*	*EF1*	*TIP41*	*PTB1*	*TUA1*
***T. aurantii*** **infestation at 48 h**										
ΔCT	*ACTIN1*	*EF1*	*PTB1*	*TUA1*	*SAND1*	*UBC1*	*CLATHRIN1*	*TIP41*	*TBP*	*GAPDH1*
BestKeeper	*ACTIN1*	*EF1*	*PTB1*	*TUA1*	*UBC1*	*SAND1*	*TBP*	*CLATHRIN1*	*GAPDH1*	*TIP41*
Normfinder	*ACTIN1*	*PTB1*	*EF1*	*TUA1*	*SAND1*	*CLATHRIN1*	*UBC1*	*TIP41*	*TBP*	*GAPDH1*
Genorm	*EF1 | TUA1*	*PTB1*	*SAND1*	*UBC1*	*ACTIN1*	*CLATHRIN1*	*TIP41*	*TBP*	*GAPDH1*	
Recommended comprehensive ranking	*ACTIN1*	*EF1*	*PTB1*	*TUA1*	*SAND1*	*UBC1*	*CLATHRIN1*	*TBP*	*TIP41*	*GAPDH1*
***E. onukii*** **infestation in the time interval from 12 h to 72 h**										
ΔCT	*UBC1*	*GAPDH1*	*EF1*	*TIP41*	*SAND1*	*TBP*	*TUA1*	*CLATHRIN1*	*PTB1*	*ACTIN1*
BestKeeper	*SAND1*	*EF1*	*TIP41*	*GAPDH1*	*CLATHRIN1*	*UBC1*	*PTB1*	*TBP*	*ACTIN1*	*TUA1*
Normfinder	*GAPDH1*	*UBC1*	*EF1*	*TIP41*	*SAND1*	*TBP*	*TUA1*	*CLATHRIN1*	*PTB1*	*ACTIN1*
Genorm	*GAPDH1 | UBC1*	*EF1*	*TIP41*	*SAND1*	*TBP*	*TUA1*	*CLATHRIN1*	*PTB1*	*ACTIN1*	
Recommended comprehensive ranking	*GAPDH1*	*UBC1*	*EF1*	*SAND1*	*TIP41*	*TBP*	*CLATHRIN1*	*TUA1*	*PTB1*	*ACTIN1*
***E. onukii*** **infestation at 96 h**										
ΔCT	*PTB1*	*TBP*	*GAPDH1*	*UBC1*	*ACTIN1*	*SAND1*	*CLATHRIN1*	*TIP41*	*EF1*	*TUA1*
BestKeeper	*EF1*	*GAPDH1*	*ACTIN1*	*SAND1*	*UBC1*	*PTB1*	*CLATHRIN1*	*TBP*	*TUA1*	*TIP41*
Normfinder	*PTB1*	*TBP*	*GAPDH1*	*UBC1*	*ACTIN1*	*SAND1*	*CLATHRIN1*	*TIP41*	*EF1*	*TUA1*
Genorm	*PTB1 | TBP*	*GAPDH1*	*UBC1*	*ACTIN1*	*CLATHRIN1*	*SAND1*	*EF1*	*TIP41*	*TUA1*	
Recommended comprehensive ranking	*PTB1*	*TBP*	*GAPDH1*	*UBC1*	*ACTIN1*	*EF1*	*SAND1*	*CLATHRIN1*	*TIP41*	*TUA1*
***E. onukii*** **infestation in the time interval from 120 h to 144 h**										
ΔCT	*TIP41*	*EF1*	*TBP*	*UBC1*	*GAPDH1*	*SAND1*	*CLATHRIN1*	*ACTIN1*	*TUA1*	*PTB1*
BestKeeper	*UBC1*	*GAPDH1*	*EF1*	*CLATHRIN1*	*TIP41*	*ACTIN1*	*TBP*	*SAND1*	*PTB1*	*TUA1*
Normfinder	*TIP41*	*EF1*	*UBC1*	*TBP*	*GAPDH1*	*SAND1*	*CLATHRIN1*	*ACTIN1*	*TUA1*	*PTB1*
Genorm	*TIP41 | TBP*	*EF1*	*UBC1*	*GAPDH1*	*SAND1*	*CLATHRIN1*	*ACTIN1*	*TUA1*	*PTB1*	
Recommended comprehensive ranking	*TIP41*	*EF1*	*UBC1*	*TBP*	*GAPDH1*	*CLATHRIN1*	*SAND1*	*ACTIN1*	*TUA1*	*PTB1*
***E. obliqua*** **regurgitant treatment in the time interval from 1.5 h to 3 h**										
ΔCT	*TIP41*	*SAND1*	*ACTIN1*	*CLATHRIN1*	*TBP*	*PTB1*	*UBC1*	*EF1*	*TUA1*	*GAPDH1*
BestKeeper	*TBP*	*ACTIN1*	*PTB1*	*UBC1*	*TIP41*	*CLATHRIN1*	*SAND1*	*EF1*	*TUA1*	*GAPDH1*
Normfinder	*ACTIN1*	*TIP41*	*SAND1*	*PTB1*	*TBP*	*CLATHRIN1*	*UBC1*	*EF1*	*TUA1*	*GAPDH1*
Genorm	*TIP41 | TBP*	*SAND1*	*CLATHRIN1*	*EF1*	*ACTIN1*	*PTB1*	*UBC1*	*TUA1*	*GAPDH1*	
Recommended comprehensive ranking	*TIP41*	*TBP*	*ACTIN1*	*SAND1*	*PTB1*	*CLATHRIN1*	*UBC1*	*EF1*	*TUA1*	*GAPDH1*
***E. obliqua*** **regurgitant treatment at 6 h**										
ΔCT	*TBP*	*CLATHRIN1*	*SAND1*	*UBC1*	*TIP41*	*ACTIN1*	*PTB1*	*GAPDH1*	*EF1*	*TUA1*
BestKeeper	*GAPDH1*	*UBC1*	*TIP41*	*ACTIN1*	*SAND1*	*CLATHRIN1*	*PTB1*	*EF1*	*TBP*	*TUA1*
Normfinder	*TBP*	*SAND1*	*UBC1*	*CLATHRIN1*	*ACTIN1*	*TIP41*	*PTB1*	*GAPDH1*	*EF1*	*TUA1*
Genorm	*CLATHRIN1 | TIP41*	*UBC1*	*TBP*	*SAND1*	*ACTIN1*	*EF1*	*PTB1*	*GAPDH1*	*TUA1*	
Recommended comprehensive ranking	*TBP*	*CLATHRIN1*	*UBC1*	*TIP41*	*SAND1*	*GAPDH1*	*ACTIN1*	*PTB1*	*EF1*	*TUA1*
***E. obliqua*** **regurgitant treatment in the time interval from 12 h to 48 h**										
ΔCT	*SAND1*	*CLATHRIN1*	*TBP*	*PTB1*	*GAPDH1*	*ACTIN1*	*TIP41*	*UBC1*	*EF1*	*TUA1*
BestKeeper	*SAND1*	*ACTIN1*	*TBP*	*CLATHRIN1*	*PTB1*	*GAPDH1*	*TIP41*	*UBC1*	*EF1*	*TUA1*
Normfinder	*SAND1*	*TBP*	*CLATHRIN1*	*PTB1*	*GAPDH1*	*ACTIN1*	*TIP41*	*UBC1*	*EF1*	*TUA1*
Genorm	*SAND1 | TBP*	*CLATHRIN1*	*PTB1*	*TIP41*	*UBC1*	*GAPDH1*	*ACTIN1*	*EF1*	*TUA1*	
Recommended comprehensive ranking	*SAND1*	*TBP*	*CLATHRIN1*	*PTB1*	*ACTIN1*	*GAPDH1*	*TIP41*	*UBC1*	*EF1*	*TUA1*

**Table 5:** Summary of treatments and results.

No.	Treatments			Recommended RGs for each treatment
	Names	Organs	Conditions	
1	Circadian rhythm of different tissues	Leaf	All test period	*CsUBC1*, *CsCLATHRIN1*
		Stem	All test period	*CsTUA1*, *CsSAND1*
		Root	All test period	*CsSAND1, CsUBC1*
2	JA treatment	2nd leaves	0.5–1.5 h	*CsCLATHRIN1*, *CsUBC1*
			3–6 h	*CsGAPDH1*, *CsTIP41*
			12–48 h	*CsCLATHRIN1*, *CsGAPDH1*
			All test period	*CsCLATHRIN1*, *CsGAPDH1*
3	*T. aurantii* infestation	2nd leaves	6–24 h	*CsACTIN1*, *CsUBC1*
			48 h	*CsACTIN1*, *CsEF1*
			All test period	*CsACTIN1*, *CsUBC1*
4	*E. onukii* infestation	2nd leaves	12–72 h	*CsGAPDH1, CsUBC1*
			96 h	*CsPTB1*, *CsTBP*
			120–144 h	*CsTIP41*, *CsEF1*
			All test period	*CsGAPDH1, CsUBC1*
5	Mechanical damage and *E.obliqua* regurgitant treatment	2nd leaves	1.5–3 h	*CsTIP1*, *CsTBP1*
			6 h	*CsTBP*, *CsCLATHRIN*
			12–48 h	*CsSAND1*, *CsTBP*
			All test period	*CsSAND1*, *CsTBP*

#### Stem

GeNorm identified *SAND1* and *TIP41* as the most stable RGs in all test periods (from 0:00 am to 22:00 pm) (Table 4). NormFinder and the ΔCt method identified *TUA1* and *CLATHRIN1* as the most stable RGs. BestKeeper identified *TUA1*, *CLATHRIN1* and *SAND1* as the top three RGs. However, all four methods identified *GAPDH1* as the most unstable RG (Table 3). According to the results from RefFinder, the stability ranking of RGs from the most to the least was as follows: *TUA1* > *SAND1* > *CLATHRIN1* > *UBC1* > *TIP41* > *PTB1* > *ACTIN1* > *TBP* > *EF1* > *GAPDH1*. Based on the ranking of the RGs by RefFinder, *TUA1* and *SAND1* were identified as the best combination for normalizing the diurnal expression in the stem (Table 5).

#### Root

NormFinder and the ΔCt method identified *UBC1* and *SAND1* as the most stable RGs, and *ACTIN1* as the least stable RG in all test period (from 0:00 am to 22:00 pm) (Table 3). GeNorm identified *SAND1* as the most stable RG. BestKeeper identified *TIP41* as the most stable RG. According to the results of RefFinder, the stability ranking of RGs from the most to the least was as follows: *SAND1* > *UBC1* > *TBP* > *TIP41* > *CLATHRIN1* > *PTB1* > *GAPDH1* > *EF1* > *TUA1* > *ACTIN1*. The results of the geNorm analysis revealed that all V values were below 0.15 (Fig. 2). Thus, *SAND1* and *UBC1* were identified as the best combination for normalizing the gene diurnal expression in roots (Table 5).

### JA treatment

GeNorm, NormFinder and the ΔCt method identified *CLATHRIN1*, *GAPDH1* and *UBC1* as the top three stable RGs in all test periods (from 0.5 h to 48 h) (Table 3). BestKeeper identified *SAND1*, *PTB1* and *TIP41* as the top three stable RGs. All four methods identified *TUA1* as the most unstable RG (Table 3). According to the results of RefFinder, the stability ranking of RGs from the most to the least was as follows: *CLATHRIN1* > *GAPDH1* > *UBC1* > *TIP41* > *PTB1* > *SAND1* > *TBP* > *ACTIN1* > *EF1* > *TUA1*. The results of the geNorm analysis revealed that all V values were below 0.15 (Fig. 2). Thus, *CLATHRIN1* and *GAPDH1* were identified as the best combination for normalizing JA-treated leaves. With further analysis, RefFinder identified *CLATHRIN1* and *UBC1* as the best combination for JA treatment in the time interval from 0.5 h to 1.5 h, *GAPDH1* and *TIP41* as the best combination in the time interval from 3 h to 6 h, and *CLATHRIN1* and *GAPDH1* as the best combination in the time interval from 12 h to 48 h (Tables 4 and 5).

### *T. aurantii* infestation

NormFinder and ΔCt identified *ACTIN1* and *UBC* as the most stable 2 of the 10 RGs in all test periods (from 6 h to 48 h) (Table 4). BestKeeper ranked *ACTIN1* and *EF1* as the top two stable RGs. GeNorm ranked *ACTIN1* and *TBP* as the top two RGs. According to the results of RefFinder, the stability ranking of RGs from the most to the least was as follows: *ACTIN1* > *UBC1* > *GAPDH1* > *CLATHRIN1* > *TBP* > *EF1* > *PTB1* > *SAND1* > *TIP41* > *TUA1* (Table 3). The results of the geNorm analysis revealed that almost all V values were below 0.15 (Fig. 2). Thus, *ACTIN1* and *UBC1* were identified as the best combination for normalizing *T. aurantii*-infested leaves. With further analysis, RefFinder identified *ACTIN1* and *UBC1* as the best combination in the time interval from 6 h to 24 h, *ACTIN1* and *EF1* as the best combination at 48 h (Tables 4 and 5).

### *E. onukii* infestation

The GeNorm, NormFinder and ΔCt methods identified *GAPDH1* and *UBC1* as the most stable 2 of the 10 RGs, while *PTB1* was the least stable RG in all test periods (from 12 h to 144 h) (Table 3). BestKeeper identified *EF1*, *GAPDH1* and *CLATHRIN1* as the top three stable RGs. According to the results of RefFinder, the stability ranking of RGs from the most to the least was as follows: *UBC1* > *GAPDH1* > *EF1* > *TIP41* > *SAND1* > *CLATHRIN1* > *TBP* > *TUA1* > *ACTIN* > *PTB1*. The results of the geNorm analysis revealed that all V values were below 0.15 (Fig. 2). Thus, *UBC1* and *GAPDH1* were identified as the best combination for normalizing *E. onukii*-infested leaves. With further analysis, RefFinder identified *GAPDH1* and *UBC1* as the best combination in the time interval from 12 h to 72 h, *PTB1* and *TBP* as the best combination at 96 h, *TIP41* and *EF1* as the best combination in the time interval from 120 h to 144 h (Tables 4 and 5).

### Mechanical damage and *E. obliqua* regurgitant treatment

GeNorm, NormFinder and the ΔCt method identified *SAND1* and *TBP1* as the most stable 2 of the 10 RGs, while *TUA1* was the least stable RG in all test periods (from 1.5 h to 48 h) (Table 3). BestKeeper identified *ACTIN1*, *CLATHRIN1* and *TBP* as the top three stable RGs. According to the results of RefFinder, the stability ranking of RGs from the most to the least was as follows: *SAND1* > *TBP* > *CLATHRIN1* > *PTB1* > *ACTIN1* > *TIP41* > *UBC1* > *EF1* > *GAPDH1* > *TUA1*. The results of geNorm revealed that all V values were below 0.15 (Fig. 2). Thus, *SAND1* and *TBP1* were identified as the best combination for normalizing regurgitant-treated leaves. With further analysis, RefFinder identified *TIP41* and *TBP* as the best combination in the time interval from 1.5 h to 3 h, *TBP* and *CLATHRIN1* as the best combination at 6 h, and *SAND1* and *TBP* as the best combination in the time interval from 12 h to 48 h (Tables 4 and 5).

### Validation of proposed RGs

**Figure 3:**
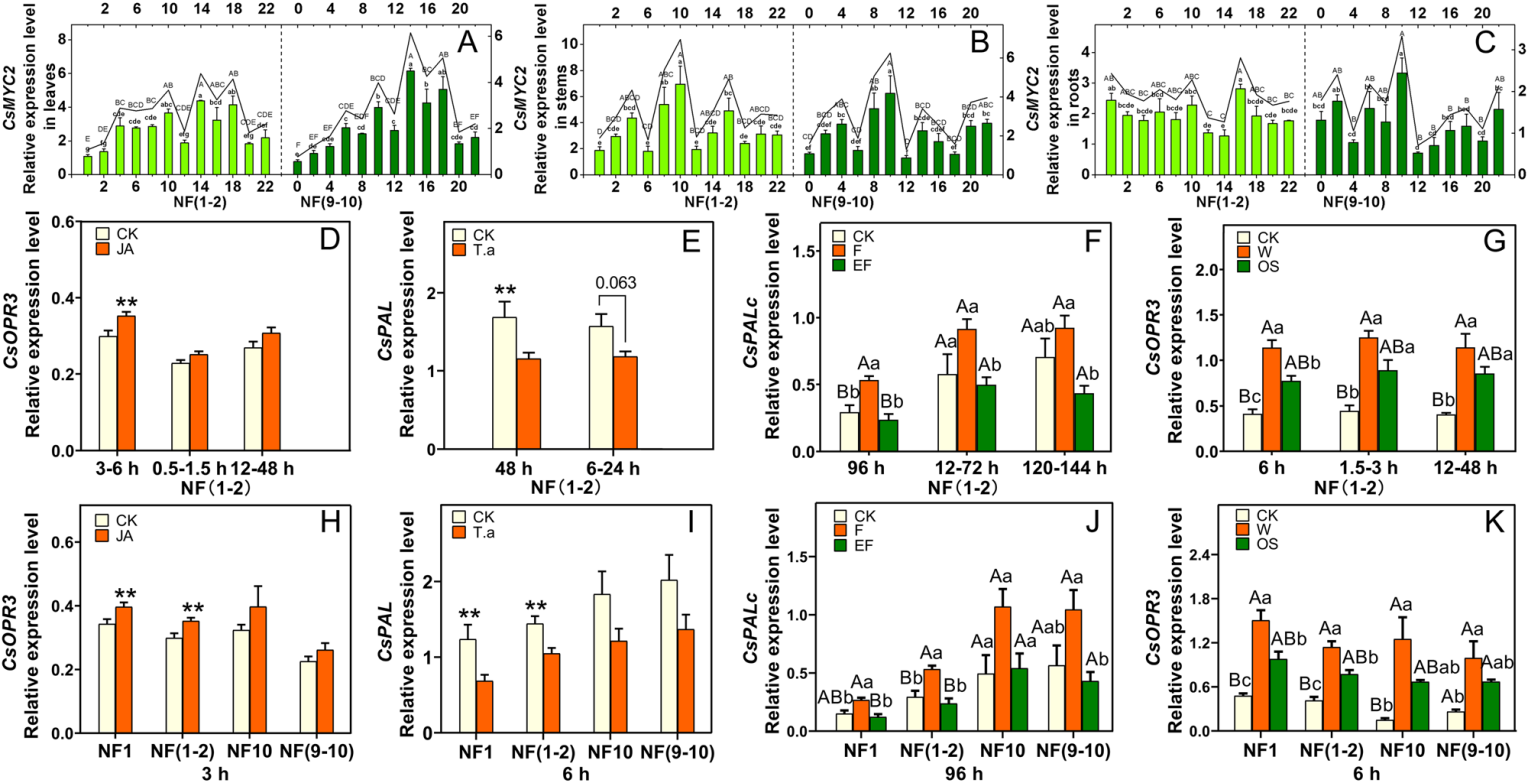
Validation of the gene stability measure. Expression profiles of *CsMYC2*, *CsOPR3*, *CsPAL* and *CsPALc* under different experimental conditions using different RGs. (**A**) Diurnal expression profile of *CsMYC2* in leaves, NF (1–2) were *UBC1* and *CLATHRIN1*, NF (9–10) were *TIP41* and *PTB1*; (**B**) Diurnal expression profile of *CsMYC2* in stems, NF (1–2) were *TUA1* and *SAND1*, NF (9–10) were *EF1* and *GAPDH1*; (**C**) Diurnal expression profile of *CsMYC2* in roots, NF(1–2) were *SAND1* and *UBC1*, NF (9–10) were *TUA1* and *ACTIN1*; (**D**) Expression profile of *CsOPR3* at 3 h normalized with the best combination (*GAPDH1* and *TIP41*) at 3 h, the best combination (*CLATHRIN1* and *UBC1*) at 0.5–1.5 h, and the best combination (*CLATHRIN1* and *GAPDH1*) at 12–48 h RGs under JA treatment; (**E**) Expression profile of *CsPAL* at 48 h normalized with the best combination (*ACTIN1* and *EF1*) at 48 h, and the best combination (*ACTIN1* and *UBC1*) at 6–24 h RGs under *T. aurantii* infestation; (**F**) Expression profile of *CsPALc* at 96 h normalized with the best combination (*PTB1* and *TBP*) at 96 h, the best combination (*GAPDH1* and *UBC1*) at 12–72 h, and the best combination (*TIP41* and *EF1*) at 120–144 h under *E. onukii* infestation; (**G**) Expression profile of *CsOPR3* at 6 h normalized with the best combination (*TBP1* and *CLATHRIN1*) at 6 h, the best combination (*TIP41* and *TBP*) at 1.5–3 h, and the best combination (*SAND1* and *TBP*) at 12–48 h RGs under *E. obliqua* infestation; (**H**) Expression profile of *CsOPR3* normalized with the stable and unstable RGs at 3 h under JA treatment. NF1 was *GAPDH1*, NF (1–2) were *GAPDH1* and *TIP41*, NF10 was *ACTIN1*, NF (9–10) were *TUA1* and *ACTIN1*; (**I**) Expression profiles of *CsPAL* normalized with the stable and unstable RGs at 6 h under *T. aurantii* infestation. NF1 was *ACTIN1*, NF (1–2) were *ACTIN1* and *UBC1*, NF10 was *TUA1*, NF (9–10) were *PTB1* and *TUA1*; (**J**) Expression profile of *CsPALc* normalized with the stable and unstable RGs at 96 h under *E. onukii* infestation. NF1 was *PTB1*, NF (1–2) were *PTB1* and *TBP*, NF10 was *TUA1*, NF (9–10) were *TIP41* and *TUA1*; (**K**) Expression profile of *CsOPR3* normalized with the stable and unstable RGs at 6 h under *E. obliqua* infestation. NF1 was *TBP*, NF (1–2) were *TBP* and *CLATHRIN1*, NF10 was *TUA1*, NF (9–10) were *EF1* and *TUA1*; Data are means ± SE. One-way ANOVA (Tukey’s test) was used to analyze significant difference among treatments (**A~C,F,G,J,K**); different letters indicate significant differences among treatments (lowercase letters, *P* < 0.05; uppercase letters, *P* < 0.01). Two samples were compared by using Student’s *t*-test (D, E, H, I); **P < 0.01.

*CsMYC2* was chosen as the target gene to validate the rationality of the recommended RGs used in diurnal expression analysis (Fig. 3A–C). The expression level of *CsMYC2* in leaves at 14:00 pm was significantly higher than that in the time period from 0:00 am to 12:00 am (NF 9–10, F = 14.098, P = 0.000; P = 0.000; P = 0.000; P = 0.000; P = 0.000; P = 0.000) and that at 16:00 pm, 20:00 pm and 22:00 pm (NF 9–10, F = 14.098, P = 0.000; P = 0.000; P = 0.000) when normalized with the two unstable RGs, *TIP41* and *PTB1* (NF 9–10); these expression level trends were quite similar to that normalized with the combination of *UBC1* and *CLATHRIN1* (NF 1–2, F = 10.169, P = 0.000; P = 0.000; P = 0.003; P = 0.003; P = 0.005; P = 0.000), except for 10:00 am (NF 1–2, F = 10.169, P = 0.138) (Fig. 3A); the expression level of *CsMYC2* in leaves at 4:00 am was significantly higher than that at 0:00 am and 2:00 am when normalized with the combination of *UBC1* and *CLATHRIN1* (NF 1–2, F = 10.169, P = 0.000; P = 0.002), but no significant differences were detected when normalized with the combination of *TIP41* and *PTB1* (NF 9–10, F = 14.098, P = 0.141; P = 0.485) (Fig. 3A). The expression level of *CsMYC2* in stem at 10:00 am was significantly higher than that at the time period from 0:00 am to 6:00 am and from 12:00 am to 22:00 pm when normalized either with the combination of *TUA1* and *SAND1* (NF 1–2, F = 3.743, P = 0.000; P = 0.003; P = 0.019; P = 0.000; P = 0.003; P = 0.008; P = 0.002; P = 0.030; P = 0.001) or with the combination of *EF1* and *GAPDH1* (NF 9–10, F = 6.969, P = 0.000; P = 0.001; P = 0.005; P = 0.000; P = 0.000; P = 0.005; P = 0.000; P = 0.005; P = 0.006), except for 16:00 pm (NF 1–2, F = 3.734, P = 0.383; NF 9–10, F = 6.969, P = 0.000); however, the expression level of *CsMYC2* in stem at 16:00 pm was significantly higher than that at 12:00 am and 18:00 pm when normalized with the combination of *TUA1* and *SAND1* (NF 1–2, F = 3.734, P = 0.030; P = 0.023), and no significant differences were detected when normalized with the combination of *EF1* and *GAPDH1* (NF 9–10, F = 6.969, P = 0.145; P = 0.256) (Fig. 3B). The expression level of *CsMYC2* at 16:00 pm in root was significantly higher than that at4:00 am, 12:00 am, 14:00 pm, 20:00 pm and 22:00 pm when normalized with the most stable combination of *SAND1* and *UBC1* (NF 1–2, F = 3.610, P = 0.013; P = 0.000; P = 0.000; P = 0.002; P = 0.003;), but the expression level of *CsMYC2* at 16:00 pm has no significant differences with that at all the time points (NF 9–10, F = 3.972, P = 0.521; P = 0.080; P = 0.464; P = 0.179; P = 0.604; P = 0.173; P = 0.360; P = 0.789; P = 0.525; P = 0.200), except for 10:00 am(NF 9–10, F = 3.972, P = 0.001), when normalized with the most unstable combination of *TUA1* and *ACTIN1* (NF 9–10) (Fig. 3C).

*CsOPR3* was chosen as the target gene to validate the rationality of the recommended RGs used in exogenous application of JA (Fig. 3D,H). When the best combination of the time interval from 3 h to 6 h, *GAPDH1* and *TIP41* (NF 1–2, F = 1.426, P = 0.028) was used for normalization, the expression level of *CsOPR3* in JA-treated leaves was significantly higher than that in the control at 3 h, but no significant difference was found when normalized with the best combination of the time interval from 0.5 h to 1.5 h, *CLATHRIN1* and *UBC1* (NF 1–2, F = 0.163, P = 0.091) or 12 h to 48 h, *CLATHRIN1* and *GAPDH1* (NF 1–2, F = 0.599, P = 0.126) (Fig. 3D). When the most appropriate RG–*GAPDH1* (NF 1, F = 0.023, P = 0.037) or the best combination of *GAPDH1* and *TIP41* (NF 1–2, F = 1.426, P = 0.028) of the time interval from 3 h to 6 h was used for normalization, the expression level of *CsOPR3* in JA-treated leaves at 3 h was significantly higher than that in the control, but no significant difference was found when normalized with the combination of the two unstable RGs, *TUA1* and *ACTIN1* (NF 9–10, F = 0.138, P = 0.204), or with the most unstable RG (NF 10, F = 3.888, P = 0.259) (Fig. 3H).

*CsPAL* was chosen as the target gene to validate the rationality of the recommended RGs used in *T. aurantii* infestation (Fig. 3E,I). When the best combination at 48 h, *ACTIN1* and *EF1* (NF 1–2, F = 2.458, P = 0.047), was used for normalization, the expression level of *CsPAL* in treated leaves at 48 h was significantly higher than that in control, but no significant difference was found when normalized with the most stable combination of the time interval from 6 h to 24 h, *ACTIN1* and *UBC1* (NF 1–2, F = 2.921, P = 0.063) (Fig. 3E). When the most appropriate RG–*ACTIN* (NF 1, F = 0.116, P = 0.041) or the best combination of *ACTIN1* and *UBC1* (NF 1–2, F = 0.245, P = 0.030) of the time interval from 6 h to 24 h was used for normalization, the expression level of *CsPAL* in treated leaves at 6 h was significantly higher than that in control, but no significant difference was found when normalized with the most unstable combination of *PTB1* and *TUA1* (NF 9–10, F = 0.820, P = 0.141) or with the most unstable RG (NF 10, F = 2.355, P = 0.120) (Fig. 3I).

*CsPALc* was chosen as the target gene to validate the rationality of the recommended RGs used in *E. onukii* infestation (Fig. 3F,J). When the best combination of *PTB1* and *TBP* at 96 h was used for normalization, the expression level of *CsPALc* at 96 h in pre-pregnant female-infested leaves was significantly higher than that of pregnant female-infested leaves (NF 1–2, F = 13.471, P = 0.002) and control leaves (F = 13.471, P = 0.008), but a relatively slight difference between pre-pregnant female-infested leaves and pregnant female-infested leaves was found when normalized with the combination of the two stable RGs in 12–72 h, *GAPDH1* and *UBC1* (NF 1–2, F = 4.838, P = 0.040) or in 120–144 h, *TIP41* and *EF1* (NF 1–2, F = 5.934, P = 0.018) (Fig. 3F). When the most appropriate RG–*PTB1*, or the most stable combination of *PTB1* and *TBP* at 96 h was used for normalization, the expression level of *CsPALc* at 96 h in pre-pregnant female-infested leaves was significantly higher than that of pregnant female-infested leaves (NF 1, F = 10.566, P = 0.005; NF 1–2, F = 13.471, P = 0.002) and control leaves (NF 1, F = 10.566, P = 0.017; NF 1–2, F = 13.471, P = 0.008), but a relatively slight difference between pregnant female-infested leaves and pre-pregnant female-infested leaves was found when normalized with the most unstable combination, *TIP41* and *TUA1* (NF 9–10, F = 4.938, P = 0.037), and no significant difference was found when normalized with the most unstable RG (NF 10, F = 4.769, P = 0.072) (Fig. 3J).

*CsOPR3* was chosen as the target gene to validate the rationality of the recommended RGs used in *E. obliqua* regurgitant treatment (Fig. 3G,K). When the best combination at 6 h, *TBP* and *CLATHRIN1* was used for normalization, the expression level of *CsOPR3* at 6 h in wounding leaves was significantly higher than that of regurgitant-treated leaves (NF 1–2, F = 32.921, P = 0.015) and intact leaves ((NF 1–2, F = 32.921, P = 0.000), but no significant difference between regurgitant-treated leaves and wounding leaves was found when normalized with the combination of the most two stable RGs in 1.5–3 h, *TIP41* and *TBP* ((NF 1–2, F = 23.023, P = 0.051) or in 12–48 h, *SAND1* and *TBP* (NF 1–2, F = 14.784, P = 0.176) (Fig. 3G). When the most appropriate RG–*TBP* (NF 1), or the most stable combination of *TBP* and *CLATHRIN1* (NF 1–2) at 6 h was used for normalization, the expression level of *CsOPR3* at 6 h in wounding leaves was significantly higher than that of regurgitant-treated leaves (NF 1, F = 26.647, P = 0.023; NF 1–2, F = 32.921, P = 0.015) and intact leaves (NF 1, F = 26.647, P = 0.001; NF 1–2, F = 32.921, P = 0.000), but no significant difference between regurgitant-treated leaves and wounding leaves was found when normalized with the most unstable combination, *EF1* and *TUA1* (NF 9–10, F = 7.557, P = 0.277) or with the most unstable RG (NF 10, F = 10.295, P = 0.117) (Fig. 3K).

## Discussion

Normalizing results with one or more appropriate internal RGs is a simple and popular method for controlling error in qRT-PCR assays. To date, a few housekeeping genes have been rigorously identified and used as RGs in tea plants under abiotic stresses, such as cold, barrenness, drought, photoperiod and exogenous application of plant hormones (auxin, ABA, GA, IAA, MeJA and SA)^25,26,28,32–34^, leaf developmental stages and even different organs^26,35^. These results demonstrate that identifying appropriate RGs for target gene expression analysis under different experimental conditions is an essential prerequisite for developing a qPCR assay of tea plants. To the best of our knowledge, the present study is the first to define the proper RGs for qRT-PCR analysis in tea plants under infestations of different herbivorous pests and their related biotic stresses.

In the present study, ten candidate RGs were selected from those already identified as stably expressed RGs with high efficiency in tea molecular studies (Table 1). Previously, *CsACINT1* was identified as one of the most unstable RGs under different experimental manipulations, such as different organs, cold or photoperiod treatment of leaves and shoots, diurnal expression in leaves, auxinole and lanolin treatment^28^. In the current study, our results showed that *CsACINT1* was ranked as one of the five most unstable RGs for diurnal variation of different organs, JA-treated leaves, infestation of *E. onukii*, and mechanical damage plus *E. obliqua* regurgitant; however, this gene was determined as the best RG in *T. aurantii* infested leaves (Table 4). Similarly, *CsACINT1* was found to be the most stably expressed RG in tea plants under Fe stress and in different organs^33^. *CsUBC1* was identified as the most stable RG in almost all treatments, except for *E. obliqua* regurgitant treatment, while *CsUBC1* was identified as the suitable RG when tea plants were under Mn stress^24^. *CsTUA1* was ranked as the most unstable RG for tea plants across most of our experimental conditions, except for diurnal expression in stems (Table 4), while previous results revealed that *CsTUA1* was the most stable RG for damage stresses of tea shoots. *CsTBP* was identified as one of the top two appropriate RGs for qRT-PCR analysis in hormonal stimuli tea leaf samples by GeNorm and NormFinder^26^, which includes ABA, GA, IAA, MeJA and SA. However, among the 10 RGs tested in this study, *CsTBP* was recommended as the seventh stable RG in JA stimuli samples, and *CsGAPDH1* and *CsCLATHRIN1* were recommended as the best RG combination for JA treatment (Table 4). The main reason for the difference is probably because different proposed RGs were adopted to rank the order. The results described above indicate, unsurprisingly, that no RG has been found to exhibit perfectly stable transcript accumulation in tea plants across different experimental conditions, even the already identified stable RGs.

The stability of the same RG varies with different plant species under diverse experimental conditions. *TIP41-like* protein (*TIP41*) was appraised as the best RG in different stages during development of bamboo (*Phyllostachys edulis*), reproductive stages of rapeseed (*Brassica napus*)^36^, and cucumber (*Cucumis sativus*) subjected to abiotic stresses and growth regulators^37^. Our results verified that *TIP41* was the second most stable RG in JA-treated leaves in the time interval from 3 h to 6 h and the most stable RG in tea leaves infested by *E. onukii* in the time interval from 120 h to 144 h (Table 5). *EF1* has been proven to be an appropriate RG for normalization of flower buds at different stages of female flower bud differentiation in the English walnut (*Juglans regia*)^38^, and *EF1* was the second stable RG in tea leaves infested by *E. onukii* in the time interval from 120 h to 144 h or infested by *T. aurantii* at 48 h as well (Table 5). Similarly, *EF1-a* gene was found to perform well for aphid-infested chrysanthemum^39^, and *EF1A 2a*, *EF1A 1a1* and *EF1A 2b* were also identified as the best RG in JA-treated leaves of soybean^40^. *GAPDH*, *ACTIN* and *UBC* are the commonly used RGs for qRT-PCR analysis in varied plant, whose function is maintaining cell survival irrespective of physiological conditions^41–43^. In this study, we found that *ACTIN*, *UBC* and *GAPDH* were the top three appropriate RGs for the whole samples of *T. aurantii*-infested leaves (Table 4), but *GAPDH* and *ACTIN* were less stable in peach^44^. *CsUBC1* was also identified as an appropriate RG in almost all treatments, except for *E. obliqua* regurgitant treatment. *HbUBC2a* and *HbUBC4* were identified as the most stable RGs in Brazilian rubber trees (*Hevea brasiliensis*) when all samples were analysed together^45^, but the *UBC2* genes were not the proper RGs in soybean (*Glycine max*) and watermelon (*Citrullus lanatus*) exposed to cadmium or under abiotic stress^46,47^. Consequently, our results emphasize that the selection of reliable RGs for normalization under any given experimental design is a requirement for developing a proper qPCR assay.

Multiple RGs have been suggested for normalizing target gene expression, which will reduce the probability of biased normalization^13,48^. In the current study, our results demonstrated using multiple RGs simultaneously in qRT-PCR analysis would increase the sensitivity of gene expression in *E. onukii* infested leaves (Fig. 3J) or *E. obliqua* regurgitant treatment (Fig. 3K). Furthermore, our results suggest that if the processing time of treatment was long, the best RGs for normalization should be recommended according to the stability of the proposed RGs in different time intervals when intragroup differences were compared (Table 5; Fig. 3D–G), which would strongly increase the accuracy and sensitivity of target gene expression in tea plants under biotic stresses. However, when the differences of intergroup were compared, the RGs for normalization should keep consistent across different time points.

In summary, we screened a series of RGs to study the gene expression profile of different organs of tea plants with circadian rhythm, JA-treated tea leaves, tea leaves attacked by *T. aurantii* or *E. onukii*, and tea leaves treated with mechanical damage plus *E. obliqua* regurgitant. Our results provide a technical guidance for further study of the molecular mechanisms of tea plants under different biotic stresses.

## Methods

### Insects

The tea aphid (*Toxoptera aurantii*), the tea leafhooper (*Empoasca onukii*) and the tea looper (*Ectropis obliqua*) were caught from the experimental tea garden of the Tea Research Institute of the Chinese Academy of Agricultural Sciences (TRI, CAAS, N 30°10′, E 120°5′), Hangzhou, China. The insects were reared on the potted tea shoots in the controlled climate room at 26 ± 2 °C, 70 ± 5% rh, and a photoperiod of 14:10 h (L:D). Newly hatched larvae/nymphs were fed on tender tea shoots that were enclosed in net cages (75 × 75 × 75 cm) and kept in the room. After one generation, mixed age nymphs of *T. aurantii* were used for plant treatment. The 4th-instar *E. onukii* nymphs were collected individually and maintained in separate plastic tubes (1.5 cm wide × 9 cm high) with fresh tea stems, and then the newly molted adults were separated by sex according to morphological characteristics. One newly molted adult female and two males were kept in a plastic container (12 cm high × 7 cm diameter) with fresh tea shoots for 5 days to obtain a fully mated female. One-day-old virgin female adults were used as feeding adults, and 6-day-old fully mated females were used as pregnant females. Our biological bioassay results showed that the pre-oviposition period is 5 d, and 6-day-old fully mated females have similar food consumption to that of 1-day-old virgin females (unpublished data). Forth-instar larvae of *E. obliqua* were used for collecting regurgitants.

### Regurgitant collection

As the method proposed by Yang *et al*.^49^, regurgitant was absorbed from *E. obliqua* oral cavity with a P200 Pipetteman (Gilson, Middleton, WI, USA). The collected regurgitant was homogenized at first. The homogeneous regurgitant was centrifuged for 5 min (10,000 × g), then the supernatant was collected and stored at −80 °C until use.

### Tea plants and treatments

Longjing 43 tea plants (three-year-old) were used for experiments, which were planted individually in a plastic pot (14 cm diameter × 15 cm high), incubated in the greenhouse programmed at12-h photophase, 26 ± 2 °C, and 70–80% relative humidity. All materials were incubated under such conditions unless otherwise stated. Plants were fertilized with fertilizer once a month and irrigated once every other day. Day before processing, tea leaves were washed under the running water. Leaves in the same position but in different branches of the same tea plant were selected for each time points. Treatments were prepared as follows.

#### Different tissues in circadian rhythm

The second leaves (numbered sequentially from the most apically unfolded leaf down the stem), stems (tender internodes between the first and the second) and fibrous roots of tea plants were harvested every 2 h of a day in the autumn of 2018. Four replications were carried out.

#### Exogeneous application of JA

JA (Sigma Chemical Co., St. Louis, MO, USA) was dissolved in a small amount of ethylalcohol and made up to a concentration of 0.15 mg/mL in 50 mM sodium phosphate buffer (titrated with 1 M citric acid until pH 8). Treatments were individually sprayed with 8 mL of JA solution. Tea plants were individually sprayed with 8 mL of the buffer were used as control. Plants were treated at 10:00 am in the climate chamber. The second leaves were harvested at 0.5, 1.5, 3, 6, 12, 24 and 48 h after the start of treatment. Each treatment was replicated five times.

#### T. aurantii infestation

Fifty aphids were inoculated on the tender bud and the 1st leaves. A fine-mesh sleeve was used to cover the 2nd leaf to prevent aphid infestation and honeydew pollution. The second leaves that covered with mesh sleeves only were used as controls. The 2nd leaves were harvested at 6, 12, 24, 48 h after the start of treatment. Each treatment was replicated five times.

#### E. onukii infestation

The 2nd tender leaf was covered with a mesh sleeve into which 4 one-day-old virgin adult females or 4 six-day-old fully mated adult females that had been starved for 2 h were introduced at 9:00 pm. Plants with only their 2nd leaves covered with mesh sleeves were used as controls. Seventy-two hours after the start of treatments, *E. onukii* adults were carefully removed. Then, the 2nd leaves were harvested at 12, 24, 48, 72, 96, 120 and 144 h after the start of removal. Each treatment was replicated six times.

#### Mechanical damage plus E. obliqua regurgitant treatment

A fabric pattern wheel was used to damage tea leaves following the method described previously (2004)^50^. Each leaf was rolled 6 times, and 15 μL regurgitant was immediately painted to the puncture wounds. Deionized water in equal amounts was painted to the wounds for wounding treatment. The intact 2nd leaf was used as control. The treated and control 2nd leaves were harvested at 1.5, 3, 6, 12, 24 and 48 h after the start of treatment. Each treatment was replicated five times.

All treatments are briefly summarized below (Table 5).

### Total RNA isolation, cDNA synthesis and qPCR analysis

The TRIzol™ kit (TIANGEN, Beijing, China) was used to isolate plant total RNA according to the protocol. The ratios of A260/280 and A260/230 of isolated RNA were examined by a spectrophotometer (Nanodrop ND 1000, Wilmington, DE, USA), and their ratios ranging from 2.0 to 2.2 and 2.0 to 2.3 individually suggested a high purity. One µg of total RNA was used to synthesize the first-strand cDNA by using a PrimerScript® RT Reagent Kit (Takara, Dalian, China) according to the protocol. A five gradient dilutions of cDNA was used as a template for each treatment to create the standard curves. After reverse transcription, the synthesized cDNA was stored at −20 °C until use.

Ten candidate RGs, including *CsACTIN1*, *CsCLATHRIN1*, *CsEF1*, *CsGAPDH1*, *CsSAND1*, *CsTIP41*, *CsUBC1*, *CsPTB1*, *CsTUA1* and *CsTBP*, were chosen from previous reports for their high stability under different stresses of tea plant (Table 2). The qPCR reactions were carried out on a LightCycle® 480 Real-Time PCR System (Roche Diagnostics, Mannheim, Germany) with a 10-μl reaction system, which contains 0.5 μl forward and reverse primers (10 μM), 5 μl FastStart Essential DNA Green Master and 25 ng first-strand complementary DNA. The programs for all genes included a preliminary step at 95 °C for 10 min, 45 cycles of denaturation amplification at 95 °C for 15 s, at 60 °C for 15 s and at 70 °C for 12 s. Finally, a melting curve analysis from 60 °C to 95 °C was carried out to confirm the specificity of the PCR products. The standard curve method was used to calculate the gene relative expression level. Each sample was analyzed in triplicate.

### Validation of selected reference genes

JA and SA signaling pathways play key roles in plant defense against herbivorous insects^51,52^, and JA and SA responsive genes could be expressed upon herbivore attack or hormone stimuli^51,53^. A key transcription factor of JA signaling–*CsMYC2*, a key enzyme in the biosynthesis of JA–*CsOPR3*, two enzyme involved in the biosynthesis of SA–*CsPAL* and *CsPALc* were selected as target genes to validate the rationality of diurnal expression in different tissues, JA treatment and *E. onukii* infestation, *T. aurantii* infestation or *E. obliqua* regurgitant treatment individually. RefFinder is a comprehensive tool, which was used to determine the geometric mean of genes. Based on the geometric mean of the genes, two different normalization factors (NFs) were the lowest and the highest mean values, and a single RG was the lowest or the highest mean value. Raw Ct values were transferred to relative quantities by the ΔΔCt method.

### Data analysis

BestKeeper, geNorm, NormFinder, the ΔCt method and RefFinder were used to evaluate the stability of the candidate RGs. All the above methods can recommend the most stable RGs. While NormFinder, geNorm and the ΔCt method rely on transforming Ct values of (1 + E) ± ΔCt, original Ct values were used in RefFinder and BestKeeper. GeNorm software was used to identify the optimum number of RGs through the cut-off value. The Vn/n + 1 value means the pair-wise variation between two sequential NFs and the optimal number of RGs required for a perfect normalization. One-way ANOVA (Tukey’s test) was used to compare the differences among more than two treatments. The difference between two samples was analyzed by Student’s *t-*test.

## Supplementary information

Supplementary Information.

Dataset 1.
