# Supplementary material for: Identification and evaluation of reliable reference genes for quantitative real-time PCR analysis in tea plants under differential biotic stresses
Source: Sci Rep. 2020 Feb 12;10:2429. doi: 10.1038/s41598-020-59168-z (PMC7015943; doi:10.1038/s41598-020-59168-z)
Supplement: Supplementary file 1 — Supplementary Information. [file 41598_2020_59168_MOESM1_ESM.pdf]

Identification and evaluation of reliable reference genes for quantitative real-time PCR analysis in tea plants under differential biotic stresses

Wei Xu[1,+], Yanan Dong[1,2,+], Yongchen Yu[2,3], Yuxian Xing[2,3], Xiwang Li[2,3], Xin Zhang[2,3], Xiangjie Hou[2,3] and Xiaoling Sun[2,3,*]
[1] College of Plant Protection, Jilin Agricultural University, Changchun, China
[2] Tea Research Institute, Chinese Academy of Agricultural Sciences, Hangzhou, Zhejiang, China
[3] Key Laboratory of Tea Biology and Resources Utilization, Ministry of Agriculture, Hangzhou, Zhejiang, China
[*] To whom correspondence should be addressed:
xlsun1974@163.com/xlsun@mail.tricaas.com (XS)
[+]these authors contributed equally to this work

Supplementary Legends:

Supplementary Figure S1. The standard curve of ten candidate reference genes of leaves with circadian rhythm.

Supplementary Figure S2. The standard curve of ten candidate reference genes of stems with circadian rhythm.

Supplementary Figure S3. The standard curve of ten candidate reference genes of roots with circadian rhythm.

Supplementary Figure S4. The standard curve of ten candidate reference genes in JA-treated leaves.

Supplementary Figure S5. The standard curve of ten candidate reference genes in *T. aurantii* infested leaves.

Supplementary Figure S6. The standard curve of ten candidate reference genes in *E. onukii* infested leaves.

Supplementary Figure S7. The standard curve of ten candidate reference genes in mechanical damage and *E. obliqua* regurgitant treated leaves.

Supplementary Figure S8. Expression profiles of ten candidate reference genes for different experimental conditions. A: Expression profiles of ten candidate reference genes for leaves with circadian rhythm; B: Expression profiles of ten candidate reference genes for stems with circadian rhythm; C: Expression profiles of ten candidate reference genes for roots with circadian rhythm; D: Expression profiles of ten candidate reference genes in JA treated leaves; E: Expression profiles of ten candidate reference genes in *T. aurantii* infested leaves; F: Expression profiles of ten candidate reference genes in *E. onukii* infested leaves; G: Expression profiles of ten candidate reference genes in mechanical damage and *E. obliqua* regurgitant treatment. As shown in Fig S8A, the raw Ct values of diurnal expression in leaf RGs ranged from 16.60 (*EF1*) to 25.02 (*SAND1*). *EF1* (17.28), *ACTIN1* (18.02), *GAPDH1* (17.84) and *TUA1* (17.90) were the most abundant transcripts, reaching the threshold fluorescence peak after 18 cycles. *CLATHRIN1* (23.11), *SAND1* (23.46), *TIP41* (22.06), *PTB1* (22.85) and *TBP* (23.46) were expressed at the lowest levels. As shown in Fig S8B, the raw Ct values of diurnal expression in stem RGs ranged from 13.90 (*EF1*) to 23.43 (*TBP*). *EF1* (16.24), *ACTIN1* (16.95), *GAPDH1* (16.70) and *TUA1* (15.86) were the most abundant transcripts, reaching the threshold fluorescence peak after 18 cycles. *CLATHRIN1* (21.64), *SAND1* (22.39), *TIP41* (21.25), *PTB1* (21.74) and *TBP* (22.13) were expressed at the lowest levels. As shown in Fig S8C, the raw Ct values of diurnal expression in root RGs ranged from 17.30 (*ACTIN1*) to 27.26 (*TBP*). *EF1* (16.24), *ACTIN1* (16.78), *GAPDH1* (16.54) and *TUA1* (15.54) were the most abundant transcripts, reaching the threshold fluorescence peak after 18 cycles. *CLATHRIN1* (24.18), *SAND1* (24.01), *PTB1* (23.10) and *TBP* (23.87) were expressed at the lowest levels. As shown in Fig S8D, the raw Ct values of diurnal expression in JA treatment RGs ranged from 16.51 (*TUA1*) to 24.34 (*PTB1*). *EF1* (17.56), *ACTIN1* (17.55), *GAPDH1* (17.61), *UBC1* (18.97) and *TUA1* (17.24) were the most abundant transcripts, reaching the threshold fluorescence peak after 18 cycles. *CLATHRIN1* (22.49), *SAND1* (22.69), *PTB1* (23.10) and *TBP* (22.98) were expressed at the lowest levels. As shown in Fig S8E, the raw Ct values of diurnal expression in *T. aurantii* infestation RGs ranged from 16.74 (*EF1*) to 26.47 (*PTB1*). *EF1* (18.04), *ACTIN1* (17.91), *GAPDH1* (18.03) and *TUA1* (18.94) were the most abundant transcripts, reaching the threshold fluorescence peak after 18 cycles. *CLATHRIN1* (23.59), *SAND1* (23.78), *TIP41* (22.93), *UBC* (21.86), *PTB1* (23.91) and *TBP* (22.66) were expressed at the lowest levels. As shown in Fig S8F, the raw Ct values of diurnal expression in *E. onukii* infestation RGs ranged from 17.62 (*EF1*) to 29.58 (*PTB1*). *EF1* (19.77), *ACTIN1* (20.56), *GAPDH1* (20.44) and *TUA1* (20.87) were the most abundant transcripts, reaching the threshold fluorescence peak after 18 cycles. *CLATHRIN1* (24.71), *SAND1* (24.58), *TIP41* (23.61), *UBC* (22.51), *PTB1* (24.65) and *TBP* (25.21) were expressed at the lowest levels. As shown in Fig S8G, the raw Ct values of diurnal expression in mechanical damage and *E. oblique* regurgitant treatment RGs ranged from 16.19 (*EF1*) to 24.73 (*SAND1*). *EF1* (7.27), *ACTIN1* (18.12), *GAPDH1* (17.96) and *TUA1* (18.52) were the most abundant transcripts, reaching the threshold fluorescence peak after 18 cycles. *CLATHRIN1* (23.01), *SAND1* (23.31), *TIP41* (22.07), *UBC* (20.97), *PTB1* (22.88) and *TBP* (22.91) were expressed at the lowest levels.

Supplementary Figure S9. The melt peak of ten candidate reference genes.

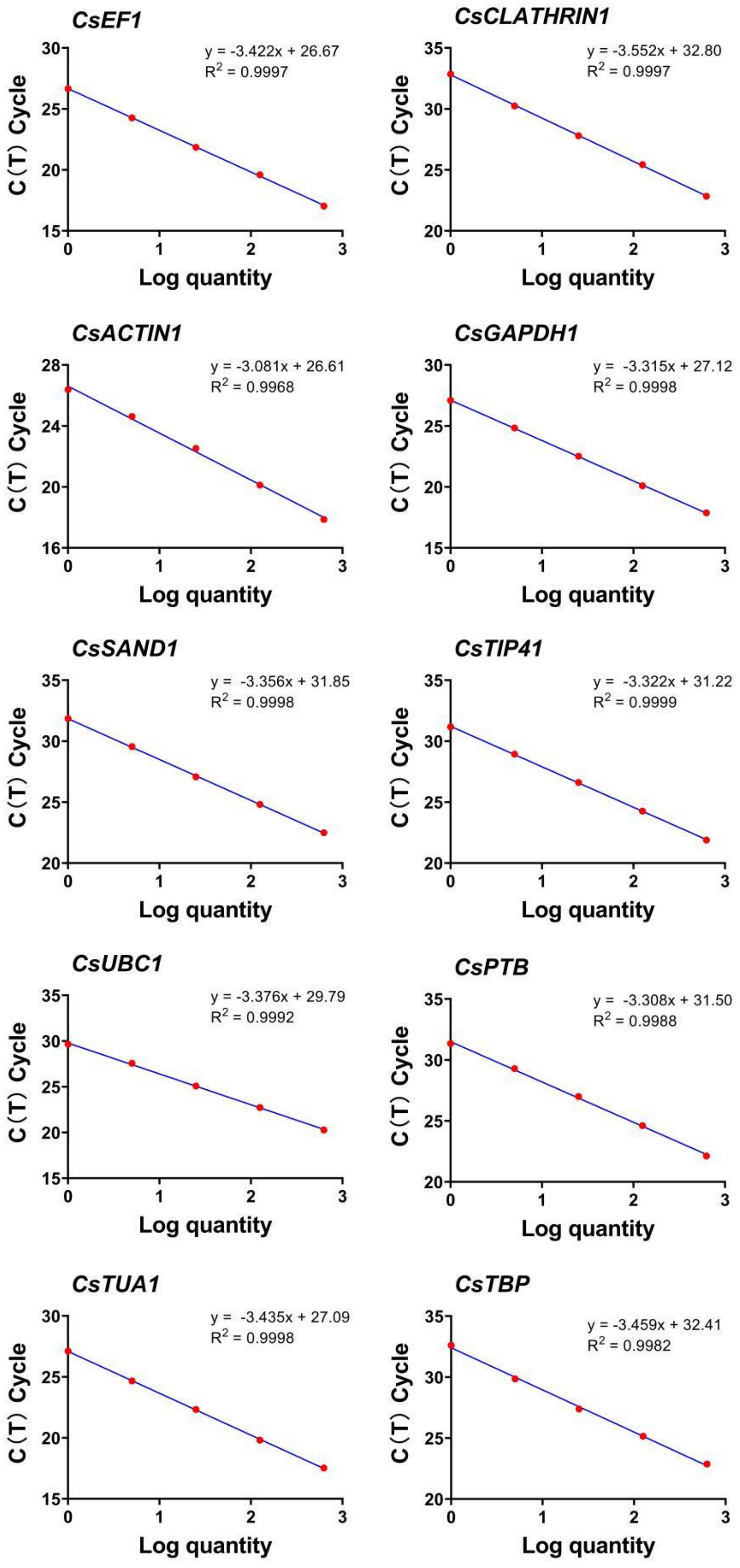

Supplementary Figure S1 of Xu et al.

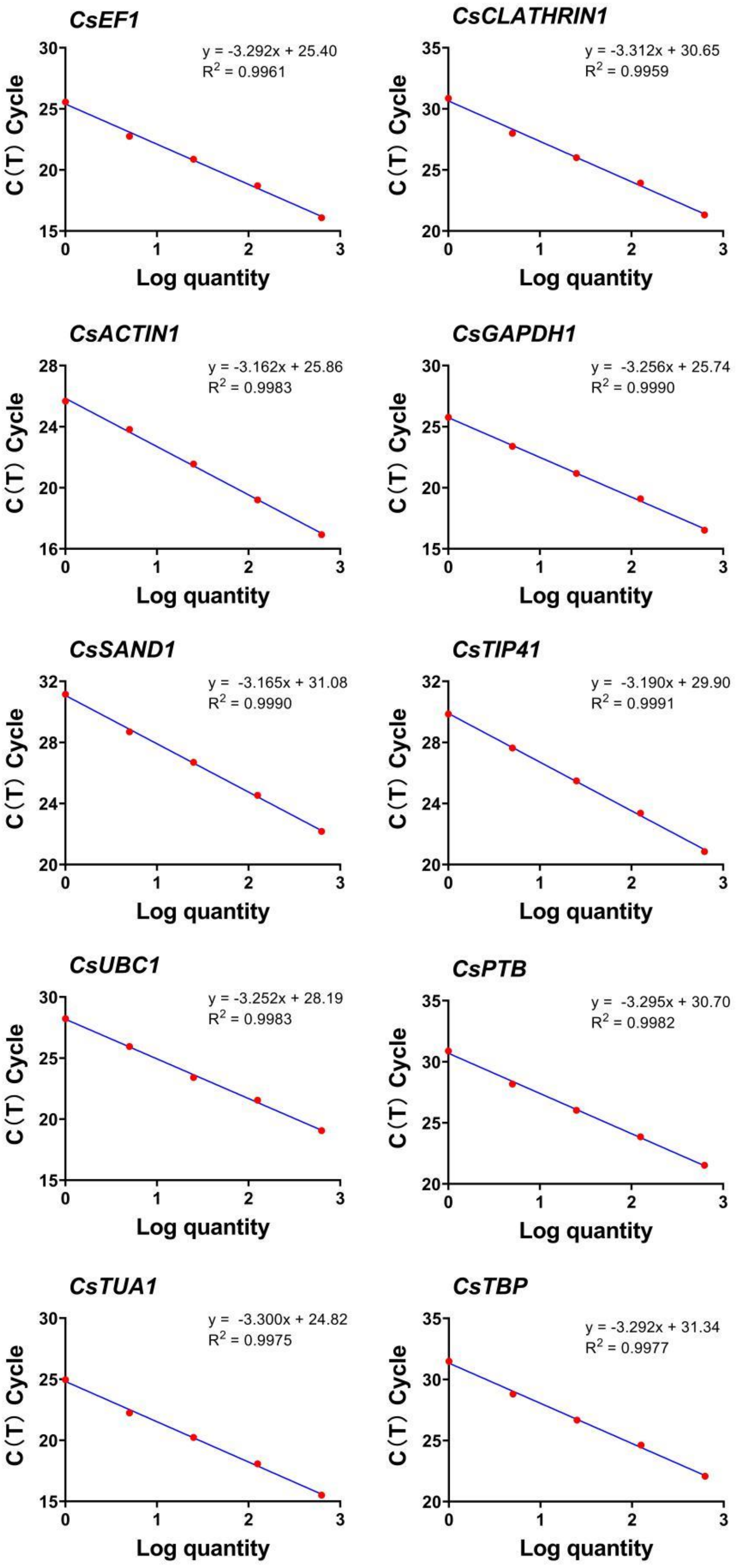

Supplementary Figure S2 of Xu et al.

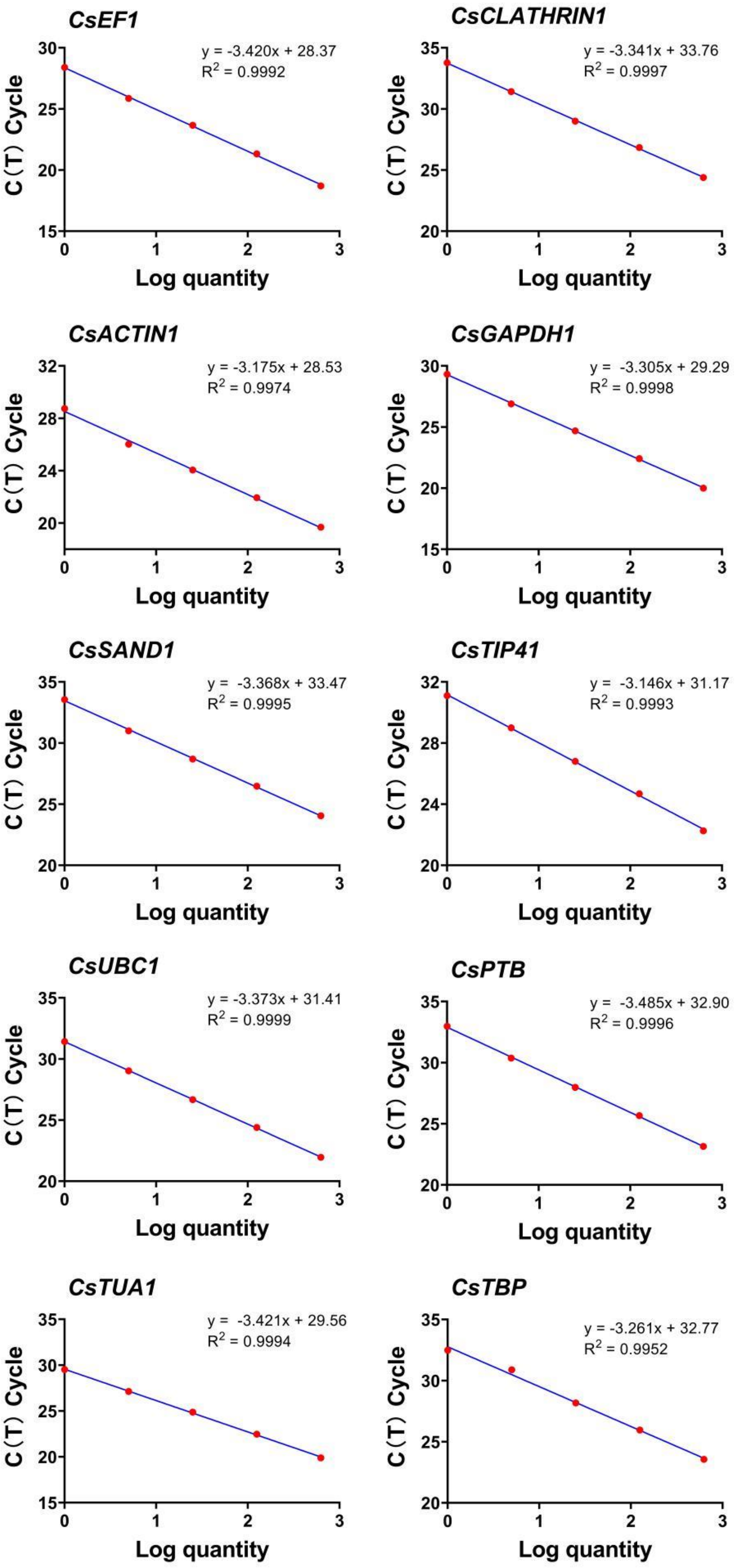

Supplementary Figure S3 of Xu et al.

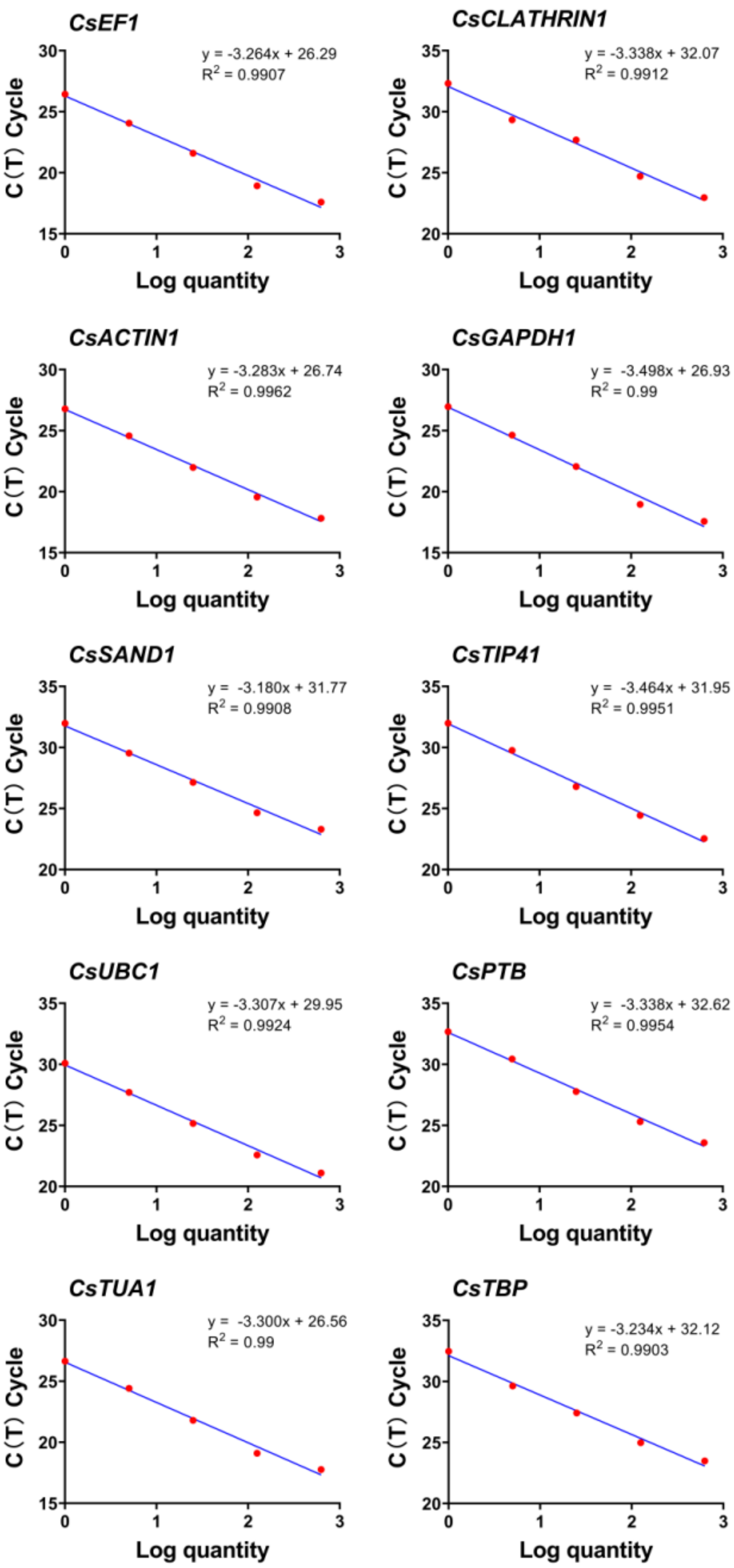

Supplementary Figure S4 of Xu et al.

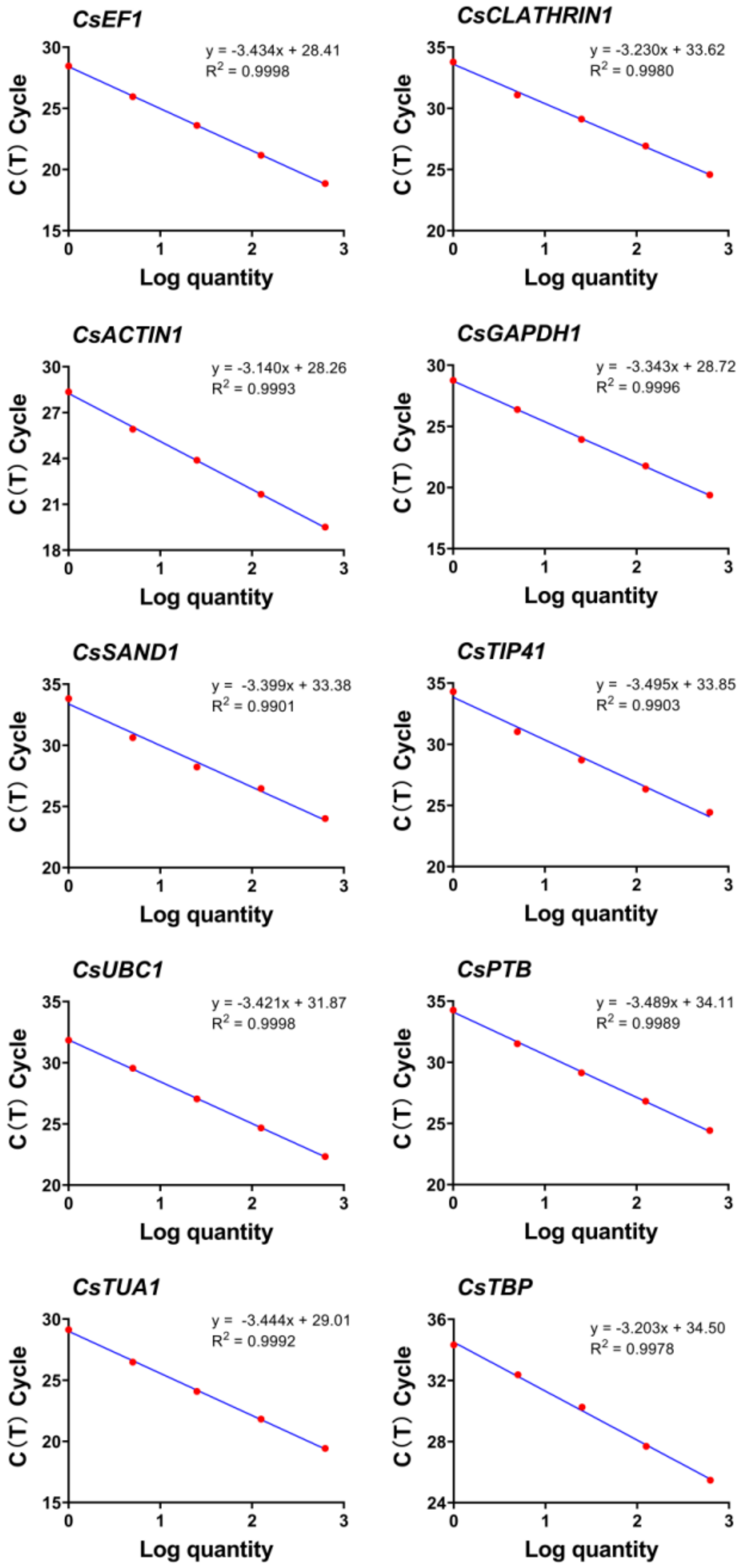

Supplementary Figure S5 of Xu et al.

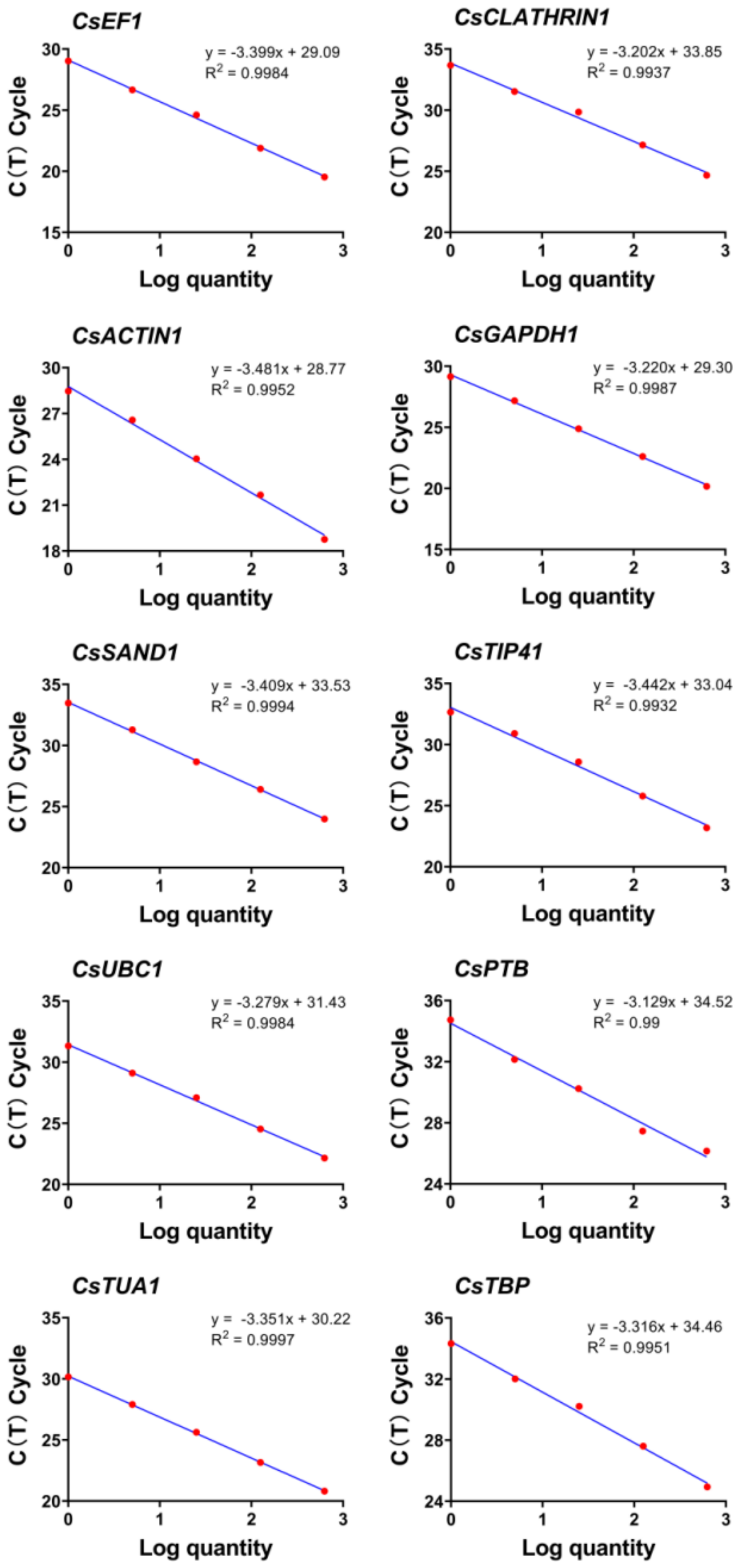

Supplementary Figure S6 of Xu et al.

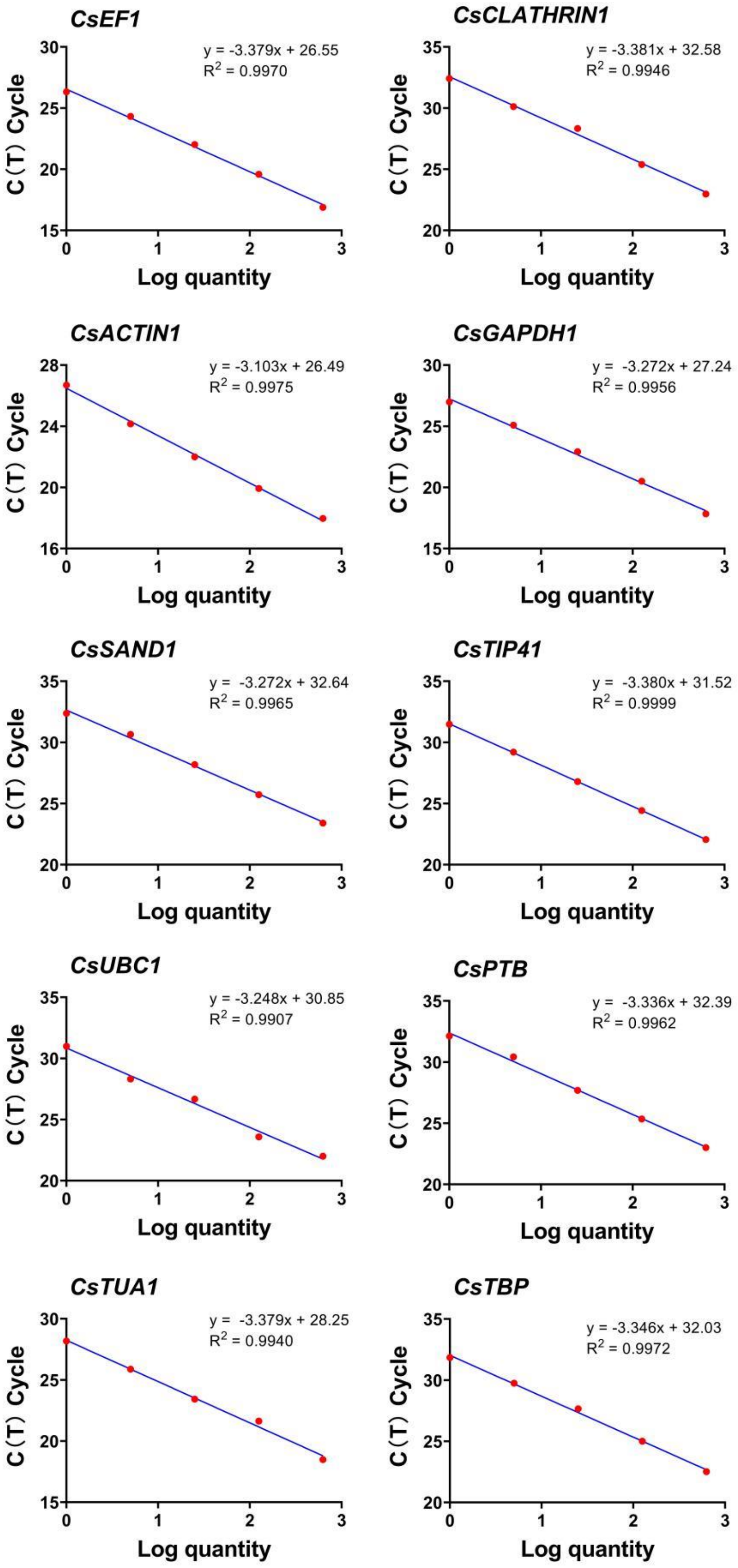

Supplementary Figure S7 of Xu et al.

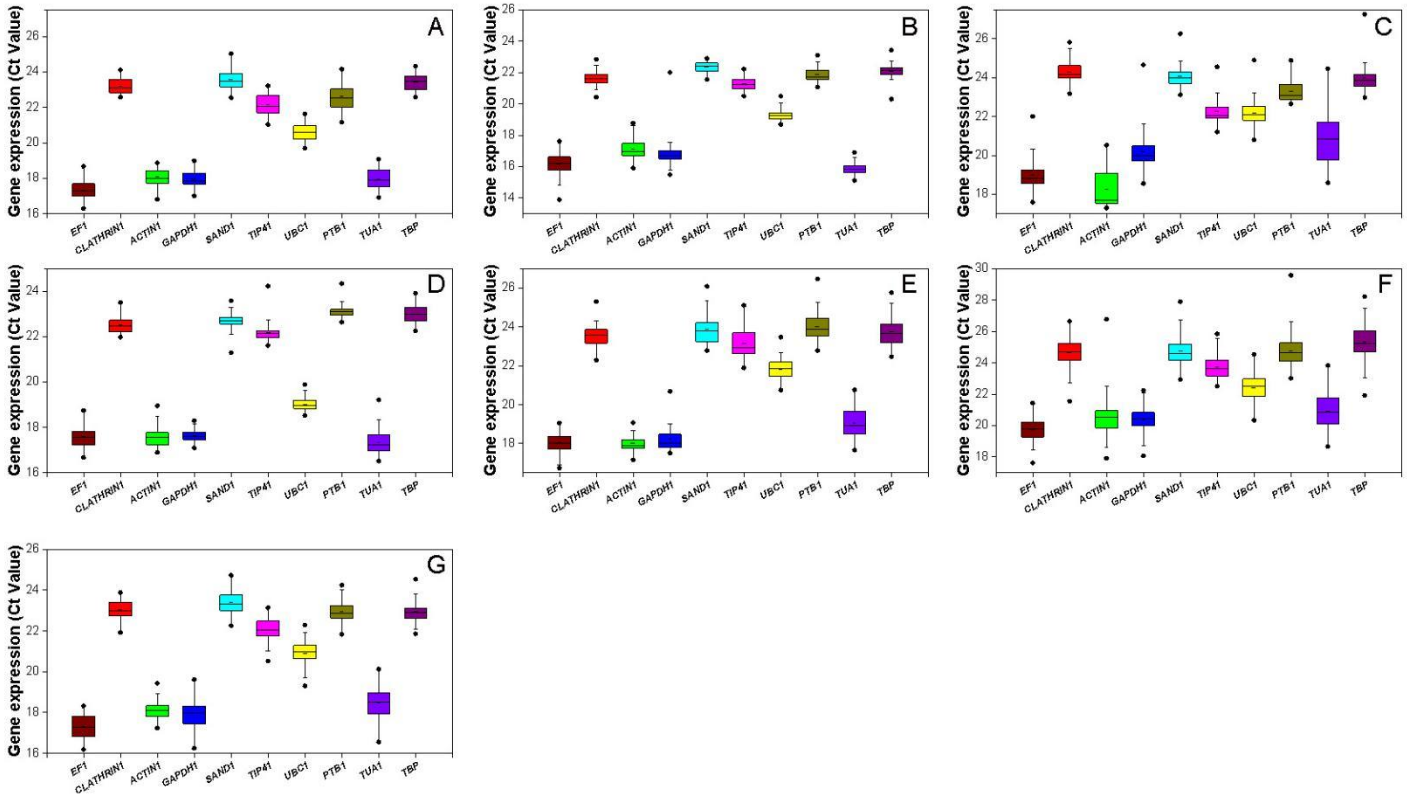

Supplementary Figure S8 of Xu et al.

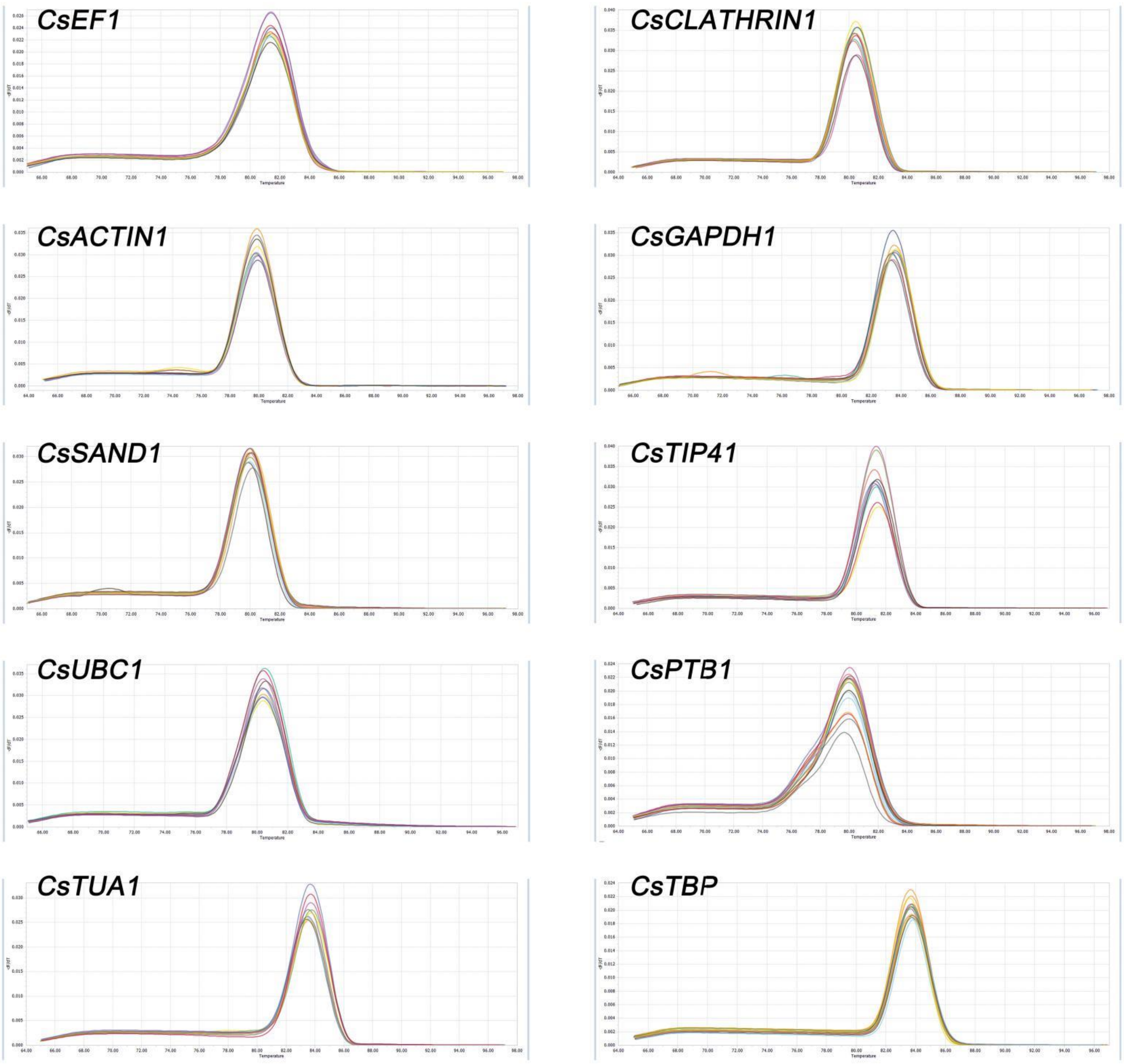

Supplementary Figure S9 of Xu et al.
